# Release of Formic Acid from Copper Formate: Hydride, Proton‐Coupled Electron and Hydrogen Atom Transfer All Play their Role

**DOI:** 10.1002/cphc.201900095

**Published:** 2019-04-29

**Authors:** Tobias F. Pascher, Milan Ončák, Christian van der Linde, Martin K. Beyer

**Affiliations:** ^1^ Institut für Ionenphysik und Angewandte Physik Universität Innsbruck Technikerstraße 25 6020 Innsbruck Austria

**Keywords:** catalysis, infrared multiple photon dissociation, hydride transfer, hydrogen atom transfer, proton-coupled electron transfer

## Abstract

Although the mechanism for the transformation of carbon dioxide to formate with copper hydride is well understood, it is not clear how formic acid is ultimately released. Herein, we show how formic acid is formed in the decomposition of the copper formate clusters Cu(II)(HCOO)_3_^−^ and Cu(II)_2_(HCOO)_5_^−^. Infrared irradiation resonant with the antisymmetric C−O stretching mode activates the cluster, resulting in the release of formic acid and carbon dioxide. For the binary cluster, electronic structure calculations indicate that CO_2_ is eliminated first, through hydride transfer from formate to copper. Formic acid is released *via* proton‐coupled electron transfer (PCET) to a second formate ligand, evidenced by close to zero partial charge and spin density at the hydrogen atom in the transition state. Concomitantly, the two copper centers are reduced from Cu(II) to Cu(I). Depending on the detailed situation, either PCET or hydrogen atom transfer (HAT) takes place.

Carbon dioxide reduction is the key step in power to fuel applications.^1^ The simplest reduced form of CO_2_ is formic acid, which has received wide attention in electrochemical carbon dioxide activation,^2^ not only as energy carrier and commodity, but also as a promising hydrogen storage material.^3^ Gas‐phase models for the formation of formic acid from CO_2_ have focused on hydrogenation at the carbon atom, either by radical abstraction in the reaction of CO_2_^−^(H_2_O)_*n*_ with thiols^4^ or by hydride transfer from metal hydrides to CO_2_,^5^, ^6^, ^7^ in both cases leading to formate. The mechanism of the release of neutral formic acid, however, is not known. We show here that formic acid release from a binary copper formate cluster proceeds by hydride transfer from formate to a metal center, followed by proton‐coupled electron transfer (PCET), with concomitant reduction of two copper centers from Cu(II) to Cu(I). One of the formate ligands is sacrificed to provide the hydride intermediate, resulting in the net reaction (1).

(1) $$2\;\mathrm{HCOO}{}^{-}\to \mathrm{HCOOH}+\mathrm{CO}{}_{2}+2\;e{}^{-}$$

Copper formate has been chosen since copper is a versatile carboxylation catalyst,^8^ and numerous gas phase studies have addressed various aspects of copper catalysis.^5^, ^6^, ^9^ Surface‐deposited size‐selected copper clusters have even been shown to catalyze the conversion of methane to methanol.^10^ Gas phase models of CO_2_ functionalization include studies with CO_2_^−^(H_2_O)_*n*_ as well as Grignard‐type metal complexes.^11^

Copper formate clusters Cu_*n*_(HCOO)_*m*_^−^ are generated by electrospray ionization and stored in the cell of a Fourier‐transform ion cyclotron resonance (FT‐ICR) mass spectrometer. Following mass selection, they are gently activated by irradiation with an infrared OPO system tuned to the antisymmetric C−O stretching vibration of the formate ligand around 1670 cm^−1^. Figure 1a shows mass spectra of mass‐selected $\mathrm{Cu}{\left(\mathrm{II}\right)}_{2}{\left(\mathrm{HCOO}\right)}_{5}^{-}$ clusters after different irradiation times. The majority of the clusters reacts by loss of formally 2 HCOO^.^ units, most likely in the form of formic acid and carbon dioxide, reaction (2), which according to our calculations lies 4.7 eV lower than loss of two formyloxyl radicals (at the B3LYP/def2TZVP level). Decomposition into smaller stoichiometric fragments, reaction (3), is a competing minor pathway. The reduced product Cu(I)_2_(HCOO)_3_^−^ undergoes decarboxylation, reaction (2).

(2) $$\mathrm{Cu}\left(\mathrm{II}\right){}_{2}\left(\mathrm{HCOO}\right){}_{5}{}^{-}\to \mathrm{Cu}\left(I\right){}_{2}\left(\mathrm{HCOO}\right){}_{3}{}^{-}+\mathrm{HCOOH}+\mathrm{CO}{}_{2}$$

(3) $$\mathrm{Cu}\left(\mathrm{II}\right){}_{2}\left(\mathrm{HCOO}\right){}_{5}{}^{-}\to \mathrm{Cu}\left(\mathrm{II}\right)\left(\mathrm{HCOO}\right){}_{3}{}^{-}+\mathrm{Cu}\left(\mathrm{II}\right)\left(\mathrm{HCOO}\right){}_{2}$$

(4) $$\mathrm{Cu}\left(I\right){}_{2}\left(\mathrm{HCOO}\right){}_{3}{}^{-}\to \mathrm{Cu}\left(I\right){}_{2}\left(\mathrm{HCOO}\right){}_{2}H{}^{-}+\mathrm{CO}{}_{2}$$

**Figure 1:**
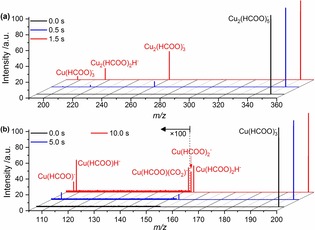
IRMPD mass spectra of a) Cu(II)_2_(HCOO)_5_^−^ and b) Cu(II)(HCOO)_3_^−^ irradiated at a) 1659 cm^−1^ and b) 1675 cm^−1^ at selected irradiation times. It should be noted that these are raw data. With the high sensitivity and resolution of the instrument, these are similar to stick spectra.

We performed extensive quantum chemical calculations to understand the mechanism of reaction (2). Since the binary Cu(II) clusters are difficult to describe, we first performed benchmark calculations against reliable thermochemical data on ${\mathrm{Cu}}^{+}{\left(H_{2}O\right)}_{n}$ and CuOH^+^(H_2_O)_*n*_ from the Armentrout laboratory,^12^ as well as single‐point CCSD calculations, see the Supporting Information (SI). The B3LYP and BMK functionals overall performed a little better than M06L, and reproduced reasonably the experimental data. The potential energy surface (PES) of the decomposition of Cu(II)_2_(HCOO)_5_^−^ is shown in Figure 2a, calculated at the B3LYP/def2TZVP level of theory, with the corresponding BMK/def2TZVP values given in parentheses.

Two paths A and B for formic acid formation, reaction (2), were found, overall exothermic by −0.62 eV at the B3LYP/def2TZVP level. As detailed in Figure 2b, Path A proceeds via TS1, TS2 and TS3, with 0.89 eV for TS2 as the highest barrier while Path B goes through TS4 and TS5, the latter lying at 1.36 eV. Both pathways compete with cluster decomposition, reaction (3), at 1.36 eV. Further decarboxylation, reaction (4), proceeds through TS6 at 0.37 eV relative to the starting point, followed by a rearrangement through TS7 to reach the $\mathrm{Cu}{\left(I\right)}_{2}{\left(\mathrm{HCOO}\right)}_{2}H^{-}$ product with *C*_2*v*_ symmetry. Overall, the B3LYP surface is relatively flat while the BMK surface shows more pronounced barriers. The discrepancy comes mostly from the binary Cu(II) species, the relative energies along the Cu(I) part of the PES are quite similar for both functionals. This indicates possible problems of the single‐reference methods to describe the interaction of two open‐shell Cu(II) centers.

**Figure 2:**
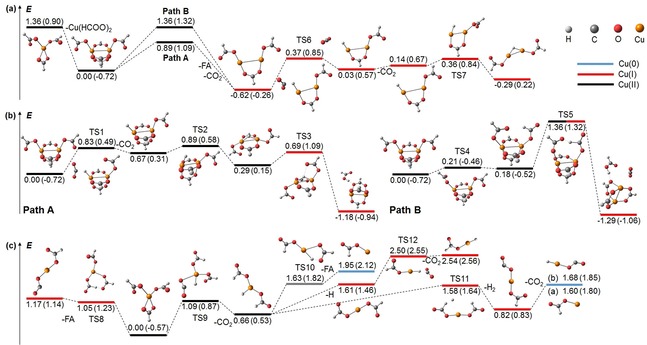
Simplified potential energy surfaces of the observed reactions. a) Decomposition of Cu(II)_2_(HCOO)_5_^−^, with formic acid (FA) elimination through Path A or B. b) Detailed PES of Path A and B. c) Decomposition of Cu(II)(HCOO)_3_^−^ resulting in Cu(0) and Cu(I) centers. Colored lines refer to the formal oxidation states of Cu. Calculated at the B3LYP/def2TZVP level of theory, with values on the BMK/def2TZVP level in parentheses shifted as to minimize the average difference between the methods. Zero‐point corrected energies are given in eV. In (b,c), complexes with weakly bound neutral products before dissociation are omitted for clarity.

While the benchmark calculations favor neither BMK nor B3LYP, only the B3LYP barriers along pathway A are consistent with experiment. The branching ratio of the decomposition reaction (3) is only 8 %, ruling out Path B with a tight transition state for both functionals. The same argument applies to TS3 calculated with BMK, lying higher than the dissociation asymptote, reaction (3). With the high‐lying TS3 in BMK, one would expect to observe the decarboxylation intermediate $\mathrm{Cu}{\left(\mathrm{II}\right)}_{2}{\left(\mathrm{HCOO}\right)}_{4}H^{-}$ , but no trace of the respective ion is visible at a noise level of 0.025 %, see Figure S1. We thus conclude that reaction (2) proceeds along pathway A as calculated with the B3LYP functional, without stabilization of the intermediate. This is plausible, given the overall exothermicity of the reaction and the relative energies of the transition states TS1≈TS2>TS3.

To get further experimental insight into the mechanistic details of the reaction, we repeated the experiment with deuterated copper formate, shown in Figure S1. The branching ratio of the dissociation reaction (3) increases from 8 % to 26 %, which allows us to derive a kinetic isotope effect KIE of 4.0 (see the SI for details). This experimental KIE indicates that the breaking of the C−H bond contributes to the rate limiting step,^13^ consistent with TS1 and TS2.

To classify the mechanisms for the two steps, we analyzed the partial charges within the CHELPG^14^ charge analysis and Mulliken spin densities along Path A, Table 1. The reactant $\mathrm{Cu}{\left(\mathrm{II}\right)}_{2}{\left(\mathrm{HCOO}\right)}_{5}^{-}$ is a singlet biradical, and only an unrestricted (UDFT) calculation yields a stable wave function. However, the spins at the two copper centers are not significantly antiferromagnetically coupled since the singlet is only 0.02 eV lower in energy than the triplet wave function (see Table S6). In TS1, the spin density stays mostly on the copper centers, and the hydrogen atom carries a significant negative charge of −0.27 e, identifying this step as a hydride transfer. Similar values are obtained for TS2. In contrast, hardly any spin density is found on neither Cu nor H atoms in TS3. Together with the negligible partial charge of −0.07 e on the H atom, the mechanism leading to formic acid is identified as proton‐coupled electron transfer (PCET).^15^, ^16^ In the products, spin density has disappeared, consistent with a closed‐shell Cu(I)_2_(HCOO)_3_^−^ product ion.

**Table 1:** Mulliken spin densities and CHELPG partial charges of Cu and H along Path A. Calculated at the B3LYP/def2TZVP level of theory; FA stands for formic acid, Cu1 and Cu2 stand for left and right copper atom, respectively, as shown in Figure 2b.

	Spin Density			Partial Charge		
Ion	Cu1	Cu2	H	Cu1	Cu2	H
Cu_2_(HCOO)_5_^−^	0.65	−0.65	0.01	1.02	1.02	−0.09
TS1	0.48	−0.66	0.16	0.89	1.04	−0.27
Cu_2_(HCOO)_4_H^−^	0.58	−0.65	0.27	0.81	1.09	−0.35
TS2	0.45	−0.59	0.22	0.95	1.20	−0.41
HCu_2_(HCOO)_4_^−^	0.41	−0.42	0.00	0.89	0.90	−0.28
TS3	0.14	−0.11	−0.05	0.87	0.87	−0.07
Cu_2_(HCOO)_3_^−^FA	0.00	0.00	0.00	0.59	0.67	0.29

Formic acid release via TS3 ultimately requires hydride, formate and two Cu(II) centers to accept the surplus electrons. The barrier from the intermediate via TS3 is only 0.40 eV, and is readily surpassed at room temperature. Reaction (1) can thus be rewritten as (1’), emphasizing these requirements.

(1’) $$\mathrm{HCOO}{}^{-}+H{}^{-}\to \mathrm{HCOOH}+2\;e{}^{-}$$

The decomposition of Cu(II)(HCOO)_3_^−^ provides additional insight into possible reactions at copper centers with relevance for the catalytic conversion of CO_2_ and hydrogen storage. Figure 1b shows IRMPD mass spectra of this species. The dominant pathway is decarboxylation, reaction (5). In the next step, the product Cu(II)(HCOO)_2_H^−^ loses a hydrogen atom, reaction (6), along with reduction of the copper center. Direct dissociation of HCOO from Cu(II)(HCOO)_3_^−^ to form $\mathrm{Cu}\left(I\right){\left(\mathrm{HCOO}\right)}_{2}^{-}$ can be ruled out: Computationally, it requires 0.82 eV more energy compared to reaching TS9. In addition, if the laser is detuned to be off‐resonance for Cu(II)(HCOO)_2_H^−^ while still being on‐resonance for Cu(II)(HCOO)_3_^−^, only Cu(II)(HCOO)_2_H^−^ is formed. This confirms that Cu(II)(HCOO)_3_^−^ does not directly decompose to Cu(I)(HCOO)_2_^−^.

(5) $$\mathrm{Cu}\left(\mathrm{II}\right)\left(\mathrm{HCOO}\right){}_{3}{}^{-}\to \mathrm{Cu}\left(\mathrm{II}\right)\left(\mathrm{HCOO}\right){}_{2}H{}^{-}+\mathrm{CO}{}_{2}$$

(6) $$\mathrm{Cu}\left(\mathrm{II}\right)\left(\mathrm{HCOO}\right){}_{2}H{}^{-}\to \mathrm{Cu}\left(I\right)\left(\mathrm{HCOO}\right){}_{2}{}^{-}+H$$

In minor amounts, Cu(I)(HCOO)(CO_2_)^−^ is observed. Due to its low intensity of less than 1 % of the total ion inventory, its precursor cannot be identified. There are two chemically plausible ways for its formation, either via formic acid elimination from Cu(II)(HCOO)_3_^−^ or H_2_ elimination from $\mathrm{Cu}\left(\mathrm{II}\right){\left(\mathrm{HCOO}\right)}_{2}H^{-}$ , reactions (7) and (7′), respectively. Other decomposition pathways include decarboxylation of Cu(I)(HCOO)_2_^−^, reaction (8), as well as two conceivable routes to the formation of Cu(0)(HCOO)^−^ (or Cu(I)(CO_2_)H^−^, see below), reactions (9) and (9′).

(7) $$\mathrm{Cu}\left(\mathrm{II}\right)\left(\mathrm{HCOO}\right){}_{3}{}^{-}\to \mathrm{Cu}\left(I\right)\left(\mathrm{HCOO}\right)\left(\mathrm{CO}{}_{2}\right){}^{-}+\mathrm{HCOOH}$$

(7’) $$\mathrm{Cu}\left(\mathrm{II}\right)\left(\mathrm{HCOO}\right){}_{2}H{}^{-}\to \mathrm{Cu}\left(I\right)\left(\mathrm{HCOO}\right)\left(\mathrm{CO}{}_{2}\right){}^{-}+H{}_{2}$$

(8) $$\mathrm{Cu}\left(I\right)\left(\mathrm{HCOO}\right){}_{2}{}^{-}\to \mathrm{Cu}\left(I\right)\left(\mathrm{HCOO}\right)H{}^{-}+\mathrm{CO}{}_{2}$$

(9) $$\mathrm{Cu}\left(\mathrm{II}\right)\left(\mathrm{HCOO}\right){}_{2}H{}^{-}\to \mathrm{Cu}\left(0\right)\left(\mathrm{HCOO}\right){}^{-}+\mathrm{HCOOH}$$

(9’) $$\mathrm{Cu}\left(I\right)\left(\mathrm{HCOO}\right)\left(\mathrm{CO}{}_{2}\right){}^{-}\to \mathrm{Cu}\left(0\right)\left(\mathrm{HCOO}\right){}^{-}+\mathrm{CO}{}_{2}$$

Cu(II)(HCOO)_3_^−^ undergoes two competing reactions (5) and (7). The PES in Figure 2c shows that direct formic acid formation (7) via TS8 requires 0.08 eV more energy than TS9 of the decarboxylation reaction (5), calculated with the B3LYP functional. The BMK values, however, are drastically in favor of decarboxylation. Already small changes of the B3LYP values would be sufficient to rationalize the observed low intensity of Cu(I)(HCOO)(CO_2_)^−^. The PES offers three competing decomposition pathways for Cu(II)(HCOO)_2_H^−^. Direct dissociation of an H atom, reaction (6), faces a similar barrier as H_2_ dissociation (7′) via TS11. The latter is an alternative to reaction (7) for formation of Cu(I)(HCOO)(CO_2_)^−^, and may be operative in hydrogen storage applications. Formic acid elimination via TS10, reaction (9), faces the highest barrier for decomposition of $\mathrm{Cu}\left(\mathrm{II}\right){\left(\mathrm{HCOO}\right)}_{2}H^{-}$ . Quantum chemistry does not provide a clear assignment of the *m*/*z* 108 peak, Cu(0)(HCOO)^−^/HCu(I)(CO_2_)^−^ are almost isoenergetic, with HCu(I)(CO_2_)^−^ only 0.08 eV higher in energy.

Reaction (5) is very interesting since the reverse reaction is formate formation, which so far was not observed with Cu(II) hydrides in the gas phase. O'Hair and co‐workers demonstrated it with Cu(I) species^6^ while He and co‐workers used Cu_2_H_2_^−^.^5^ With the reduced copper species, formate formation proceeds nearly barrierless, as corroborated in our calculated PES for the reverse of reaction (8), proceeding through TS12 in Figure 2c. With Cu(II), the reverse barrier of reaction (5) via TS9 is 0.43 eV, which does not seem prohibitive in a real‐life catalytic environment. Mechanistically, all formate decarboxylation reactions observed here proceed via hydride transfer, evidenced by the significant negative partial charge of −0.27 e≥*q*≥−0.46 e and negligible spin density on the transferred H unit, see Figures S4a, S6 for details.

It is quite intriguing to note that the reactions (7), (7′) and (9), in which Cu(II) is reduced, proceed via hydrogen atom transfer (HAT). The spin density in the transition state, displayed Figure S7, reveals that the transferred hydrogen atom is carrying a significant fraction of the spin density drawn from the Cu(II) center, with partial charges of +0.01 e≥*q*≥−0.23 e. The latter value, in comparison with the partial charges reported above for hydride transfer, however, illustrates that partial charge does not always allow to distinguish hydride from hydrogen atom transfer while spin density is a clear indicator.

In summary, we have established a mechanism for formic acid formation via PCET from two formate ligands at two Cu(II) centers. One formate ligand is sacrificed to provide the hydride required for formic acid release. HAT is operative in the decomposition of mononuclear copper formate complexes, including release of molecular hydrogen. These mechanisms may be operative at the active sites of heterogeneous copper‐hydride based catalysts.

## Experimental Section

The clusters are prepared using electrospray ionization and transferred into the cell of a 9.4 T Fourier‐transformation ion cyclotron resonance mass spectrometer which is explained in more detail elsewhere.^17^ Here, ions are mass selected and can be irradiated in the wavelength range of 4476–12000 nm by a tunable optical parametric oscillator. To trigger decomposition, the ions are vibrationally excited through photon absorption of the antisymmetric C−O stretching vibration around 1670 cm^−1^ and the fragmentation is investigated as a function of irradiation time. Further details are given in the SI. For the modelling of copper formate clusters, density functional theory (DFT) with the B3LYP and BMK functionals is used along with the def2TZVP basis set for geometry optimization. The choice of the functionals is based on benchmarking against the coupled cluster method, see Tables S1–4 in the SI. The wave function stability was tested for all calculated species. Transition states are verified through the intrinsic reaction coordinate (IRC) calculations and charge analysis is performed using the CHELPG scheme, with the Cu radius of 1.4 Å.^14^ *T*_1_ diagnostics at the CCSD/def2TZVP//B3LYP/def2TZVP level was performed (see Table S5), showing that values for ions with both one and two copper centers lie below 0.04, suggesting that single‐reference calculations might be of reasonable accuracy in most cases.^18^ However, for structures containing two Cu(II) centers, CCSD calculations had convergence issues, and no *T*_1_ value could be obtained. All calculations are carried out in *Gaussian 09*.^19^ All energies are reported with zero‐point corrections.

## Conflict of interest

The authors declare no conflict of interest.

## Supporting information

As a service to our authors and readers, this journal provides supporting information supplied by the authors. Such materials are peer reviewed and may be re‐organized for online delivery, but are not copy‐edited or typeset. Technical support issues arising from supporting information (other than missing files) should be addressed to the authors.

Supplementary
