# Supplementary material for: Release of Formic Acid from Copper Formate: Hydride, Proton‐Coupled Electron and Hydrogen Atom Transfer All Play their Role
Source: Chemphyschem. 2019 Apr 29;20(11):1420–4. doi: 10.1002/cphc.201900095 (PMC6563433; doi:10.1002/cphc.201900095)
Supplement: Supplementary file 1 — Supplementary [file CPHC-20-1420-s001.pdf]

CHEMPHYSCHEM

Supporting Information

© Copyright Wiley-VCH Verlag GmbH & Co. KGaA, 69451 Weinheim, 2019

Release of Formic Acid from Copper Formate: Hydride, Proton-Coupled Electron and Hydrogen Atom Transfer All Play their Role

Tobias F. Pascher, Milan Ončák, Christian van der Linde, and Martin K. Beyer*© 2019 The Authors. Published by Wiley-VCH Verlag GmbH & Co. KGaA. This is an open access article under the terms of the Creative Commons Attribution License, which permits use, distribution and reproduction in any medium, provided the original work is properly cited.

Experimental Methods

Isotopically enriched copper(II) formate cluster anions are introduced into the gas phase via electrospray ionisation (ESI). Isotopically enriched 98.6% Cu-63(I) oxide is dissolved at 100 mmol/l in a solution of 20% formic acid and water. The solution is heated to 70°C and stirred for several hours. For the deuterated species, isotopically enriched deuterated formic acid at 98% is used. The resulting mixture is dissolved in a 50:50 solution of methanol and water with a concentration of about 5 mmol/l. Isotopically enriched copper oxides were provided by JSC Isotope, formic acid and D-formic acid by Sigma-Aldrich (98%), HPLC grade water and methanol by Carl Roth. From the ESI source, the anions are transferred into the cell of a Bruker Apex Qe FT-ICR mass spectrometer equipped with a 9.4 T superconducting magnet. Here, the precursor ions are trapped and mass selected, followed by irradiation with an optical parametric oscillator laser system (EKSPLA NT-273-XIR), which provides tunable laser light between 4476 nm and 12000 nm at 1000 Hz.

Kinetic Isotope Effect in Experiment

In Figure S1, a typical mass spectrum after 1.5 s irradiation time of the antisymmetric C-O stretching vibration is shown for $Cu(II)_2(HCOO)_5^-$ and $Cu(II)_2(DCOO)_5^-$ as a precursor ion. The pathway leading towards formic acid formation is slightly suppressed for the deuterated case. Thus, the cluster decomposition reaction is more abundant compared to the clusters with hydrogen.

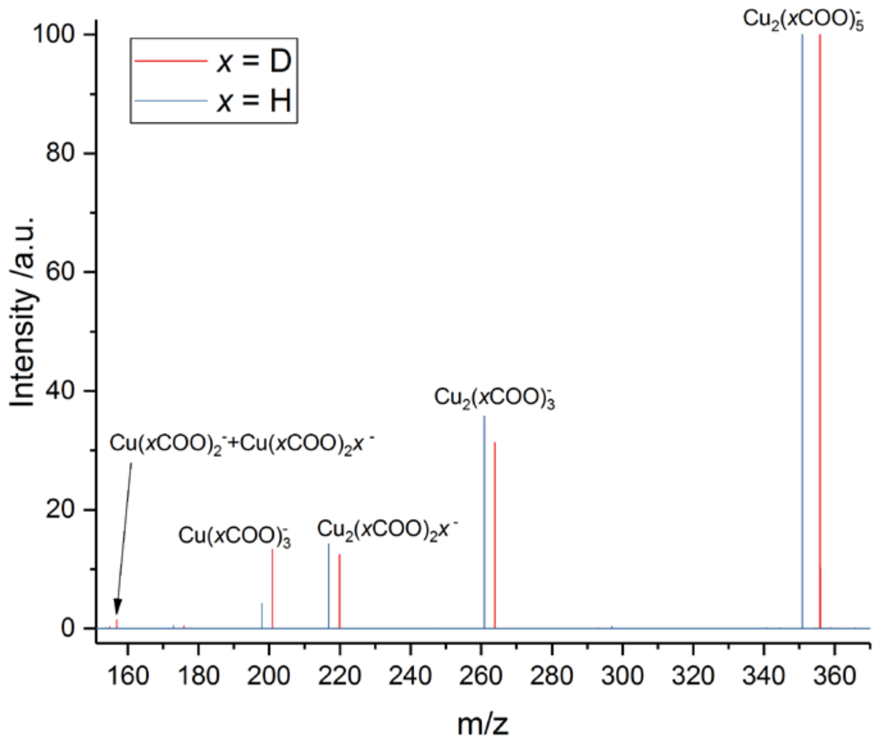

Figure S1. Mass spectrum of $Cu(II)_2(HCOO)_5^-$ irradiated at 1659 cm^{-1} compared to $Cu(II)_2(DCOO)_5^-$ irradiated at 1661 cm^{-1} after 1.5 s irradiation time.

Kinetic Isotope Effect from Branching Ratio

The kinetic isotope effect (*KIE*) is estimated by comparing branching ratios without and with deuteration, BR_H and BR_D, respectively. The branching ratio $BR_{H,D}$ into the copper dissociation channel $Cu(HCOO)_3^-$ or $Cu(DCOO)_3^-$ is calculated from the product intensities of the respective channels, which is connected with the rate coefficients *via* eq. (S1).

$$k_{dis,H}/(k_{dis,H} + k_H) = BR_H \qquad (S1)$$

Here, $k_{dis,H}$ is the rate coefficient for dissociation along the Cu-Cu bond (reaction (3)), while k_H describes the pathway leading to release of HCOOH + CO_2 (reaction (2)). $k_{dis,D}$ and k_D refer to these pathways for the deuterated species. Equation (S1) leads to

$$k_H = k_{dis,H} \bullet (1 - BR_H) / BR_H \qquad (S2)$$

The *KIE* can now be obtained by dividing k_H through k_D, eq. (S3).

$$KIE = k_H / k_D = (k_{dis,H} / k_{dis,D}) \cdot ((1- BR_H) \cdot BR_D) / (BR_H \cdot (1 - BR_D)) \qquad \text{(S3)}$$

BR_H and BR_D are obtained from ion intensities of the product channels. We assume that $k_{dis,H} / k_{dis,D}$ is close to 1, since the difference in zero point energies is 0.002 eV (B3LYP/def2TZVP) and thus negligible, and the hydrogen atom is not involved in the dissociation mechanism, reaction (3).

CID

Sustained Off Resonance Irradiation (SORI) – Collision Induced Dissociation (CID) experiments were carried out to verify that the fragmentation channels are consistent with IRMPD. An example with the precursor $Cu(II)_2(HCOO)_5^-$ is shown in Figure S2. The fragments are the same as in IRMPD, but the branching ratio of the dissociation reaction (3) is increased, consistent with the interpretation of a loose transition state for reaction (3) vs. a tight transition state for reaction (2).

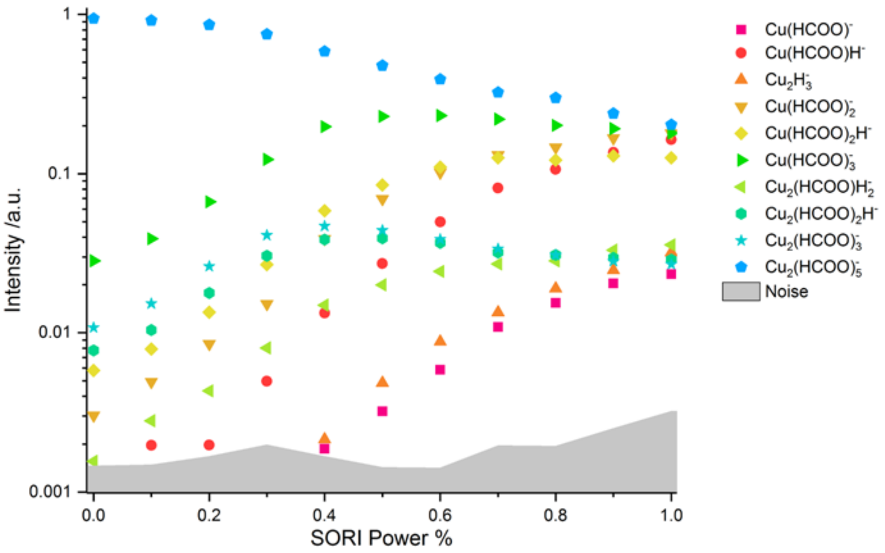

Figure S2. The decomposition of $Cu(II)_2(HCOO)_5^-$ with increasing SORI power.

Theoretical methods

Table S1 shows benchmarking of basis sets and methods for two reactions with the smallest participating molecules observed with copper formate. DFT-based methods (B3LYP, BMK, M06L and M062X) with the def2TZVP basis set agree well with higher-level methods. As a first step, all structures were optimized using B3LYP/def2TZVP. Benchmarking the observed reactions of larger systems using single point calculation involving copper formate in oxidation state +II and 0 shows that the BMK method provides good agreement with the CCSD energies with an average deviation of $\overline{|\Delta E_{CCSD} - \Delta E_i|} < 0.1$ eV (see Tables S1–3). Other methods show larger deviations with an average of $\overline{|\Delta E_{CCSD} - \Delta E_i|} \sim 0.1$–0.3 eV.

Table S4 includes water dissociation energies from Cu^+ and $CuOH^+$ in clusters with up to four water molecules. It can be seen that B3LYP and BMK methods are of similar quality in this case, with B3LYP being slightly closer to the experimental values.

The T_1 diagnostics within the CCSD method yields values of $T_1 < 0.03$ for most ions, suggesting that the single-reference approach might be of reasonable accuracy. However, T_1 values for $Cu(II)(HCOO)_2H^-$ and $Cu(0)(HCOO)^-$ ions lie between 0.03 and 0.04, suggesting possible problems with the single-reference treatment applied here. For other relevant structures, like $Cu(II)_2(HCOO)_5^-$ or TS5,

the CCSD calculations did not converge, and no T_1 value could be obtained. T_1 values of selected ions are collected in Table S5.

B3LYP/def2TZVP and BMK/def2TZVP methods were chosen in the end for geometry optimization and energy comparisons. Additionally, a denser integration grid (“Int=UltraFine” in Gaussian) was required for converging all calculations, because of a flat potential energy surface around some TSs. For every optimization, the wave functions were stabilized and, in the case of RHF/RKS instabilities, UHF/UKS calculations were performed.

The Intrinsic Reaction Coordinate (IRC) calculations are performed to verify the nature of every transition state. Hereby, for few transition states, starting points with a small offset along the normal vector of the corresponding imaginary frequency were used.

Table S1. Benchmarking of basis sets and *ab initio* methods for reaction (a), $Cu(I)(HCOO)_2^- \rightarrow Cu(I)(HCOO)H^- + CO_2$, and reaction (b), $Cu(I)(HCOO)H^- \rightarrow Cu(I)H_2^- + CO_2$. Zero-point corrected energies are given in eV. For CCSD(T), single point energies with geometry optimization and zero-point correction on the CCSD level are used. * indicates a minor imaginary frequency in the optimized geometry (In the structure of $Cu(I)(HCOO)_2^-$, calculations with BMK along with the aug-cc-pVTZ basis set, one small imaginary frequency of $-14\ cm^{-1}$ was found. Offsetting it by the corresponding amplitude results in returning to the same similar structure even with very tight convergence criteria).

Method	Basis Set	Reaction (a)	Reaction (b)
M062X	6-31++g**	1.46	1.73
	aug-cc-pVTZ	1.38	1.64
	def2SVP	1.40	1.69
	def2TZVP	1.33	1.63
B3LYP	6-31++g**	1.15	1.49
	aug-cc-pVTZ	0.98	1.33
	def2SVP	1.12	1.52
	def2TZVP	0.93	1.33
CCSD(T)	6-31++g**	1.24	1.55
	aug-cc-pVTZ	-	-
	def2SVP	1.12	1.41
	def2TZVP	-	1.49
MP2	6-31++g**	1.14	1.46
	aug-cc-pVTZ	1.10	1.41
	def2SVP	1.03	1.38
	def2TZVP	1.05	1.42
M06	6-31++g**	1.01	1.36
	aug-cc-pVTZ	0.81	1.15
	def2SVP	1.14	1.51
	def2TZVP	0.76	1.14
M06L	6-31++g**	0.89	1.24
	aug-cc-pVTZ	0.80	1.12
	def2SVP	1.10	1.41
	def2TZVP	0.75	1.08
BMK	6-31++g**	1.27	1.59
	aug-cc-pVTZ	1.13*	1.46
	def2SVP	1.26	1.61
	def2TZVP	1.08	1.44

Table S2. Relative energies (in eV) along the reaction pathway of Fig. 2a. The energies were evaluated from single point calculations in B3LYP/def2TZVP optimized structures excluding ZPE; DFT energies were shifted as to minimize the root mean square of the deviation with respect to CCSD. For CCSD, a second $Cu(I)_2(HCOO)_3^-$ isomer (b) is more stable by 0.08 eV (see Fig. S8). However, all reactions should proceed through isomer (a) as a hydride transfer reaction towards Cu would be too demanding for the closed structure of isomer (b).

Product ion	CCSD	B3LYP	BMK	M06L
$Cu(I)_2(HCOO)_3^-$ a	0.00	0.08	0.02	0.29
TS6	1.32	1.18	1.25	1.25

$Cu(I)_2(HCOO)_2H(CO_2)^-$	1.03	0.86	0.99	0.88
$Cu(I)_2(HCOO)_2H^-$ a	1.18	0.98	1.11	1.02
TS7	1.36	1.24	1.31	1.15
$Cu(I)_2(HCOO)_2H^-$ b	0.70	0.54	0.65	0.59
$Cu(I)_2(HCOO)_3^-$ b	-0.08	0.23	-0.01	0.17
$Cu(I)_2(HCOO)_3(HCOOH)(CO_2)^-$	-1.02	-0.63	-0.80	-0.84
$\overline{\lvert \Delta E_{CCSD} - \Delta E_i \rvert}$	-	0.20	0.08	0.18

Table S3. Relative energies (in eV) along the reaction pathway of Fig. 2c. The energies were evaluated from single point calculations in B3LYP/def2TZVP optimized structures excluding ZPE; DFT energies were shifted as to minimize the root mean square of the deviation with respect to CCSD.

Product ion	CCSD	B3LYP	BMK	M06L
$Cu(II)(HCOO)_3^-$	0.00	0.63	0.06	0.49
$Cu(I)(HCOO)(CO_2)^-$	1.73	1.86	1.82	2.25
TS8	2.44	1.87	2.04	1.79
TS9	1.73	1.88	1.69	1.71
$Cu(II)(HCOO)_2H^-$	1.34	1.47	1.33	1.38
TS10	2.68	2.51	2.70	2.59
$Cu(II)(HCOO)(CO_2)^-$	2.59	2.63	2.79	2.93
$Cu(I)(HCOO)_2^-$	2.34	2.56	2.41	2.48
TS11	2.71	2.56	2.63	2.48
TS12	3.71	3.60	3.66	3.43
$Cu(I)(HCOO)H^-$	3.67	3.63	3.66	3.37
$Cu(I)(HCOO)(CO_2)^-$	1.78	1.83	1.83	2.00
$Cu(0)(HCOO)^-$	2.64	2.61	2.80	2.68
$Cu(I)(CO_2)H^-$	3.05	2.83	3.01	2.83
$\overline{\lvert \Delta E_{CCSD} - \Delta E_i \rvert}$	-	0.19	0.09	0.26

Table S4. Water dissociation energies (in eV) optimized at the BMK and B3LYP levels of theory and using the CCSD(T) method in single points optimized at the B3LYP level with the B3LYP zero point energy added. The def2TZVP basis set was used. Experimental values were taken from Refs. [1].

Ion	BMK	B3LYP	CCSD(T)	Experiment
Cu^+H_2O	1.88	1.77	1.57	1.67
$Cu^+(H_2O)_2$	1.89	1.76	1.61	1.76
$Cu^+(H_2O)_3$	0.63	0.57	0.61	0.59
$Cu^+(H_2O)_4$	0.48	0.45	0.53	0.56
$CuOH^+H_2O$	2.26	2.09	1.74	1.86
$CuOH^+(H_2O)_2$	1.39	1.12	1.47	1.31
$CuOH^+(H_2O)_3$	1.01	0.79	1.01	0.93
$CuOH^+(H_2O)_4$	0.77	0.72	0.74	0.59
$\overline{\lvert \Delta E_{exp} - \Delta E_i \rvert}$	0.15	0.11	0.10	-

Table S5. Values of T_1 diagnostics for selected ions as calculated at the CCSD/def2TZVP//B3LYP/def2TZVP level of theory.

Ion	T_1 diag.
$Cu(I)_2(HCOO)_3(HCOOH)(CO_2)^-$	0.0237
$Cu(I)_2(HCOO)_3^-$	0.0259
TS6	0.0264
$Cu(I)_2(HCOO)_2H(CO_2)^-$	0.0253
$Cu(I)_2(HCOO)_2H^-$ a	0.0269
TS7	0.0266
$Cu(I)_2(HCOO)_2H^-$ b	0.0262
$Cu(II)(HCOO)_3^-$	0.0237
$Cu(II)(HCOO)_2H^-$	0.0381
$Cu(I)(HCOO)_2^-$	0.0245
TS10	0.0248
TS11	0.0263
TS12	0.0248
$Cu(I)(HCOO)H^-$	0.0260
$Cu(I)(HCOO)(CO_2)^-$	0.0259
$Cu(I)(CO_2)H^-$	0.0281
$Cu(0)(HCOO)^-$	0.0321

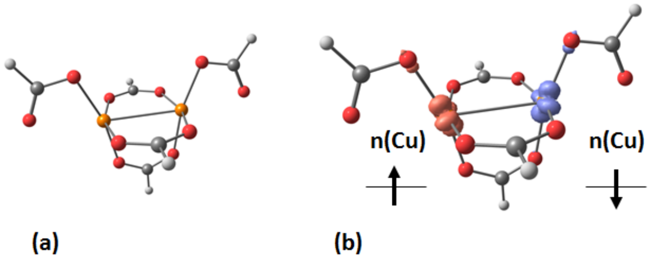

Figure S3. a) Structure of $Cu(II)_2(HCOO)_5^-$ along with its b) spin density. Calculated at the B3LYP/def2TZVP level of theory.

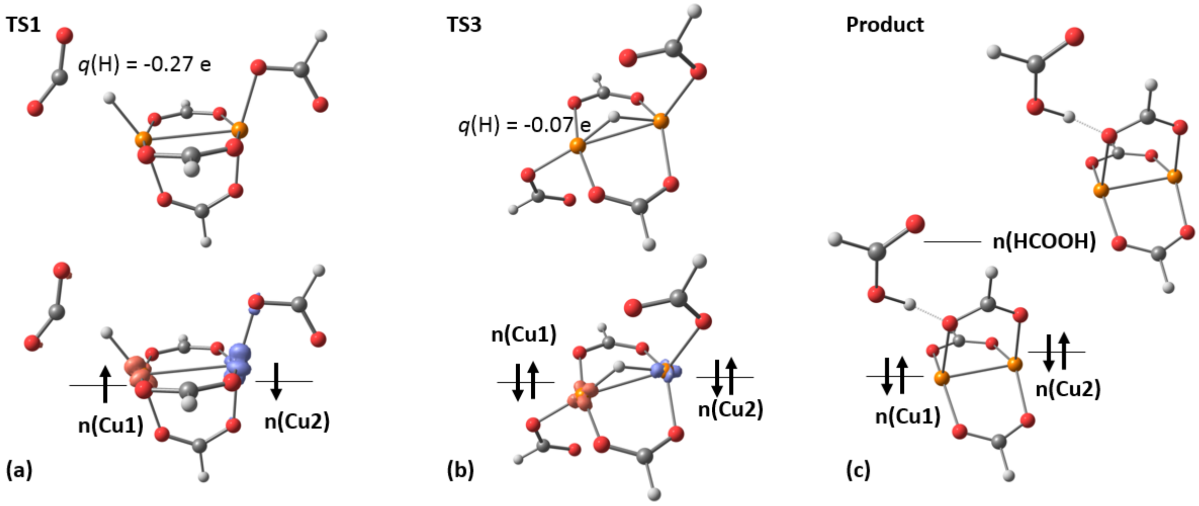

Figure S4. a) Structure of the hydride transfer in TS1, b) structure of the PCET in TS3 and c) structure of product channel following TS3. Spin densities shown in the lower part of the figure. Charge q on H is calculated using the CHELPG scheme. Calculated at the B3LYP/def2TZVP level of theory. The absence of the spin density on the H atom rules out the HAT mechanism.

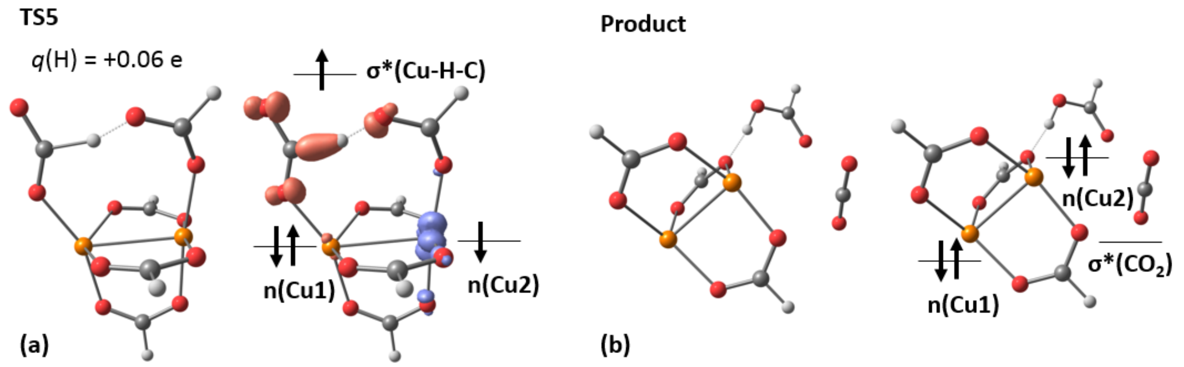

Figure S5. a) Structure for the HAT in TS5 and b) structure of its product channel. Spin densities shown on the right. Charge *q* on H is calculated using the CHELPG scheme. Calculated at the B3LYP/def2TZVP level of theory. The siginifant spin density on the H atom indicates HAT.

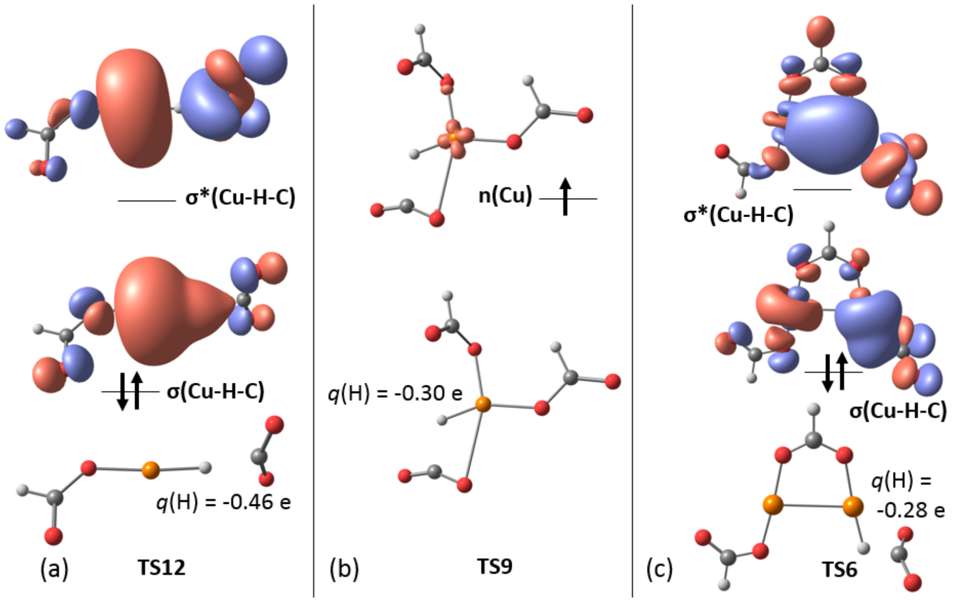

Figure S6. Structure of the hydride transfer transition states. In a), c), doubly occupied three-center *σ*(Cu-H-C) and *σ**(Cu-H-C) orbitals are shown. In b), the spin density is shown. Charge *q* on H is calculated using the CHELPG scheme. Calculated at the B3LYP/def2TZVP level of theory.

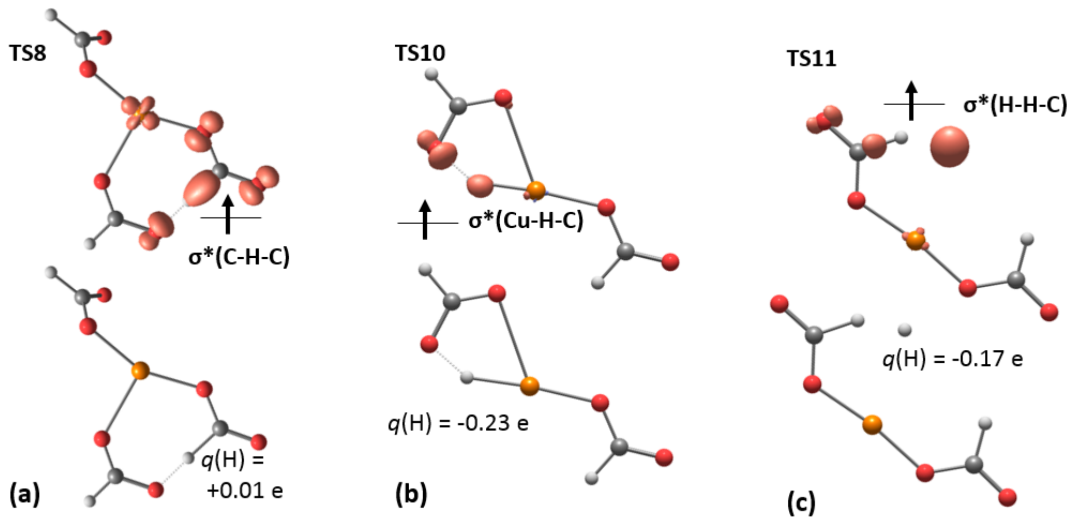

Figure S7. Structure of the HAT transition states from Figure 2c) with the CHELPG charge *q* of the transferred hydrogen and the spin density at the B3LYP/def2TZVP level of theory.

Figure S8. Structure of $Cu_2(HCOO)_3^-$ at the at the BMK/def2TZVP level of theory with isomer (a) being favorable to (b) by 0.13 and -0.02 eV using B3LYP/def2TZVP and BMK/def2TZVP, respectively.

Table S6. Relative energies (in eV) of several ions optimized in singlet and triplet spin multiplicity; calculated at the B3LYP/def2TZVP (BMK/def2TZVP) level of theory. Low values (below 0.03 eV) indicate the absence of (anti)ferromagnetic coupling between Cu ions.

Reaction	ΔE
$Cu(II)_2(HCOO)_5^-$ (M = 1) → $Cu(II)_2(HCOO)_5^-$ (M = 3)	0.02 (0.01)
TS5 (M = 1) → TS5 (M = 3)	0.02 (0.01)
TS1 (M = 1) → TS1 (M = 3)	0.02 (0.01)
$Cu(II)_2(HCOO)_4H^-$ (M = 1) → $Cu(II)_2(HCOO)_4H^-$ (M = 3)	0.02 (0.01)
TS2 (M = 1) → TS2 (M = 3)	0.19 (0.08)
$Cu(II)_2H(HCOO)_4^-$ (M = 1) → $Cu(II)_2H(HCOO)_4^-$ (M = 3)	0.38 (0.14)
$Cu(II)_2(HCOO)_5^-$ (M = 3) → $Cu(I)_2(HCOO)_3^-$ + CO_2 + HCOOH (M = 3)	2.33 (3.03)

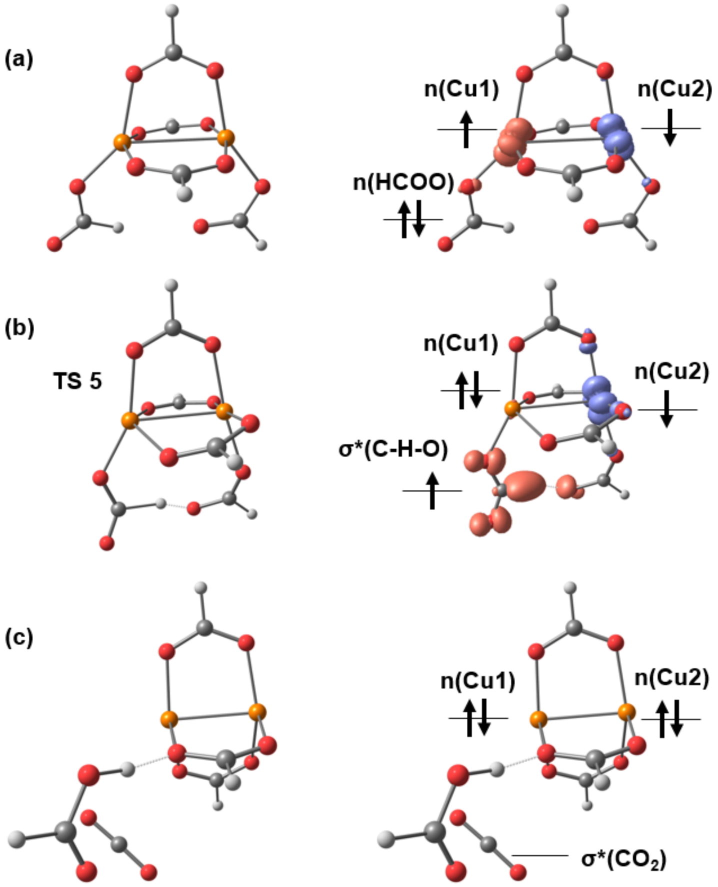

Figure S9. The molecular structure (left), along with the spin density (right) for Path B in Figure 2. a) $Cu(II)_2(HCOO)_5^-$ reactant, b) TS5, c) the dissociation complex. Calculated at the M06-2X/def2TZVP//B3LYP/def2TZVP level of theory. The spin density shows that this pathway proceeds via HAT.

Literature

[1] a) N. F. Dalleska, K. Honma, L. S. Sunderlin, P. B. Armentrout, *J. Am. Chem. Soc.* **1994**, *116*, 3519; b) A. F. Sweeney, P. B. Armentrout, *J. Phys. Chem. A* **2014**, *118*, 10210.

Cartesian coordinates of optimized ions and molecules (in Å, calculated at the BMK/def2TZVP level) along with electronic energies (in Hartree) including zero point energy

Cu2(HCO2)5-, TS4
E= -4225.421604
Cu 1.483921 -0.192247 0.061708
Cu -1.457600 0.546531 0.052528
O -1.385585 -0.923614 1.351322
C -0.484307 -1.776958 1.455531
O 0.645579 -1.773306 0.929224
O 1.295458 0.874499 1.717691
C 0.362969 1.645534 2.003065
O -0.730715 1.775587 1.419636
O -0.774370 1.758349 -1.368910
C 0.429681 2.017579 -1.557466
O 1.429190 1.471703 -1.054688
O 2.122289 -1.267725 -1.475789
C 3.335635 -1.319008 -1.125359
O 3.777695 -0.792958 -0.102006
H 4.014137 -1.875362 -1.797080
H -0.718148 -2.640994 2.097093
H 0.636612 2.838527 -2.264722
H 0.522750 2.295197 2.879013
O -2.788512 -0.065317 -1.146499
C -3.606541 -1.054170 -1.120831
O -3.739095 -1.921275 -0.286791
H -4.258774 -1.057027 -2.022292

CO2
E=-188.569891
O 0.000000 0.000000 1.153125
C 0.000000 0.000000 0.000000
O 0.000000 0.000000 -1.153125

HCu(CO2)-
E=-1828.919295
C 1.227930 1.094960 0.000000
O 1.830165 2.140664 0.000000
O 0.000000 0.825120 0.000000
Cu -0.712387 -0.963704 0.000000
H -1.349676 -2.348607 0.000000

Cu(HCO2)-
E=-1828.921167
C -1.586723 0.280363 0.000124
O -2.770438 -0.025053 -0.000165
O -0.590157 -0.505180 0.000158
Cu 1.301218 0.041065 -0.000035
H -1.330222 1.368800 0.000320

CuH(HCO2)-
E=-1829.552602
Cu -1.094481 -0.074870 -0.000004
O 2.053936 -0.864753 -0.000008
C 1.746059 0.317114 0.000014
O 0.595256 0.849323 0.000011
H 2.567888 1.074915 0.000003
H -2.497820 -0.682919 -0.000012

Cu(HCO2)3-, TS8
E=-2207.239742
o -2.491678 1.493352 0.364695
h -2.303460 0.140814 0.215952
c -2.150516 -1.127192 0.089866
o -3.151273 -1.793009 0.188577
o -0.946467 -1.414253 -0.120174
cu 0.524777 -0.251252 -0.315240
o -0.334218 1.757281 -0.197267
c -1.437124 2.162350 0.158269
o 2.363287 0.102134 -0.786129
c 3.175850 -0.057255 0.177562
o 2.921136 -0.412871 1.318018
h 4.230993 0.163898 -0.096119
h -1.561621 3.253094 0.326184

Cu(HCO2)2-
E=-2018.162858
C -2.761408 -0.319458 -0.000020
O -2.994104 0.876598 0.000008
O -1.636179 -0.915377 -0.000019
Cu -0.000021 0.000427 -0.000001
O 1.636644 0.915315 0.000014
C 2.761541 0.318771 0.000012
O 2.993547 -0.877420 0.000006
H -3.616888 -1.033022 -0.000016
H 3.617427 1.031848 0.000018

Cu(HCO2)2
E=-2018.065295
C 2.294781 -0.000132 0.000023
O 1.661402 1.083445 -0.000099
O 1.661159 -1.083557 -0.000081
Cu 0.000000 0.000137 0.000014
O -1.661416 1.083443 0.000042
C -2.294780 -0.000140 -0.000016
O -1.661146 -1.083559 0.000059
H 3.390661 -0.000248 0.000180
H -3.390660 -0.000271 0.000014

Cu(DCO2)2
E=-2018.071807
C 2.294781 -0.000132 0.000023
O 1.661402 1.083445 -0.000099
O 1.661159 -1.083557 -0.000081
Cu 0.000000 0.000137 0.000014
O -1.661416 1.083443 0.000042
C -2.294780 -0.000140 -0.000016
O -1.661146 -1.083559 0.000059
H(Iso=2) 3.390661 -0.000248 0.000180
H(Iso=2) -3.390660 -0.000271 0.000014

Cu(HCO2)3-
E=-2207.306013
Cu -0.000001 0.081576 -0.000006
O -1.157898 -1.197436 -0.898180
C -2.192677 -1.301771 -0.169664
O -2.399632 -0.709754 0.881346
H -2.954971 -2.006190 -0.561385
O -0.710725 1.845139 -0.827941
C 0.000347 2.452668 -0.000012
O 0.711139 1.844910 0.827991
H 0.000440 3.555350 0.000060
O 1.157622 -1.197705 0.898162
C 2.192392 -1.302193 0.169657
O 2.399445 -0.710199 -0.881349

H 2.954575 -2.006734 0.561375

Cu(DCO2)3-
E=-2207.315509
Cu -0.000001 0.081576 -0.000006
O -1.157898 -1.197436 -0.898180
C -2.192677 -1.301770 -0.169664
O -2.399632 -0.709753 0.881346
H(Iso=2) -2.954972 -2.006189 -0.561385
O -0.710724 1.845139 -0.827941
C 0.000348 2.452668 -0.000012
O 0.711140 1.844910 0.827991
H(Iso=2) 0.000441 3.555350 0.000060
O 1.157622 -1.197705 0.898162
C 2.192392 -1.302194 0.169657
O 2.399445 -0.710200 -0.881349
H(Iso=2) 2.954574 -2.006735 0.561375

Cu(HCO2)CO2-
E=-2017.526788
C 2.679062 -0.186798 -0.007995
O 1.671249 0.576185 0.009825
Cu -0.068126 -0.115986 0.003660
O -1.774500 -0.882152 -0.000062
C -2.830022 -0.166367 -0.004119
O -2.925884 1.047119 -0.005836
O 3.858971 0.041914 -0.007333
H -3.757265 -0.781956 -0.006211

CuH(HCO2)2-
E=-2018.695670
C 2.864439 0.193672 0.000101
O 3.978278 -0.285678 0.000143
O 1.759958 -0.461290 -0.000334
Cu 0.000000 0.182329 -0.000189
O -1.759958 -0.461289 0.000030
C -2.864440 0.193673 0.000298
O -3.978278 -0.285678 0.000499
H 2.739173 1.297235 0.000367
H -2.739175 1.297236 0.000332
H 0.000003 1.745399 -0.000307

Cu2H(HCO2)2-, Iso. 2
E=-3658.540521
H 4.797802 1.090870 0.002872
Cu 1.254581 -0.132494 -0.000482
C 3.947071 0.374574 0.001762
O 4.179995 -0.821979 0.000869
Cu -1.254589 -0.132471 -0.001439
O -2.812348 0.954463 -0.001411
C -3.947069 0.374553 0.001185
O -4.179939 -0.822010 0.003539
O 2.812325 0.954444 0.001637
H -4.797832 1.090812 0.001183
H -0.000008 -1.111781 -0.003108

TS5, *M* = 3
E=-4225.355938
cu 0.217974 -1.469838 -0.103768
cu -1.131537 0.998174 0.077569
o 0.218329 2.299014 0.724186
c 1.378719 2.542605 0.351860
h 1.721721 3.582889 0.511235
o 2.224932 1.792701 -0.173905
h 2.395792 0.380109 -0.019274
c 2.972511 -0.753612 0.109457
o 2.199351 -1.713551 0.053596
o 4.161217 -0.589069 0.245930
o -0.700541 1.536930 -1.749130
c 0.007554 0.716719 -2.393471
o 0.445083 -0.363066 -1.996140
o -1.627394 0.652312 1.935886
c -0.842947 -0.147768 2.512621
o 0.075336 -0.786222 1.995615
o -2.640593 -0.106263 -0.556203
c -2.626738 -1.359068 -0.585618
o -1.680221 -2.123447 -0.357090
h 0.247836 1.026352 -3.426855
h -1.019564 -0.271328 3.597080
h -3.591041 -1.827728 -0.853466

TS5, *M* = 1
E=-4225.356130
cu 0.208902 -1.468746 -0.114636
cu -1.124367 1.003069 0.087224
o 0.244834 2.277404 0.748657
c 1.398689 2.527764 0.361658
h 1.740917 3.567653 0.522533
o 2.240396 1.783828 -0.180321
h 2.406102 0.378014 -0.021196
c 2.972421 -0.763492 0.109943
o 2.189731 -1.715174 0.048045
o 4.162091 -0.611876 0.252782
o -0.690178 1.558170 -1.732383
c 0.010171 0.738282 -2.386085
o 0.438564 -0.349142 -2.000501
o -1.626448 0.649482 1.940642
c -0.856348 -0.167626 2.513197
o 0.052309 -0.817640 1.994167
o -2.640513 -0.086336 -0.558538
c -2.633088 -1.338793 -0.604389
o -1.691359 -2.111300 -0.384068
h 0.251990 1.056949 -3.416288
h -1.037648 -0.295505 3.596371
h -3.599383 -1.798593 -0.880275

Deuterated TS5, *M* = 1
E=-4225.370658
cu 0.208902 -1.468746 -0.114636
cu -1.124367 1.003069 0.087224
o 0.244834 2.277404 0.748657
c 1.398689 2.527764 0.361658
h(Iso=2) 1.740917 3.567653 0.522533
o 2.240396 1.783828 -0.180321
h(Iso=2) 2.406102 0.378014 -0.021196
c 2.972421 -0.763492 0.109943
o 2.189731 -1.715174 0.048045
o 4.162091 -0.611876 0.252782
o -0.690178 1.558170 -1.732383
c 0.010171 0.738282 -2.386085
o 0.438564 -0.349142 -2.000501
o -1.626448 0.649482 1.940642
c -0.856348 -0.167626 2.513197
o 0.052309 -0.817640 1.994167
o -2.640513 -0.086336 -0.558538
c -2.633088 -1.338793 -0.604389
o -1.691359 -2.111300 -0.384068
h(Iso=2) 0.251990 1.056949 -3.416288
h(Iso=2) -1.037648 -0.295505 3.596371
h(Iso=2) -3.599383 -1.798593 -0.880275

Cu2H(HCO2)2-, Iso. 1
E=-3658.523790
O -3.873536 0.341606 -0.000071
H -4.020700 -1.663054 -0.000027
C -3.364076 -0.765640 -0.000028

O -2.126152 -1.069461 0.000010
Cu -0.843135 0.290192 0.000005
O 0.314890 1.782948 -0.000007
C 1.561616 1.841261 -0.000035
H 1.981307 2.862940 -0.000058
O 2.385812 0.913944 -0.000040
Cu 2.124469 -1.010181 0.000037
H 2.087363 -2.526224 0.000122

Cu2(HCO2)3-, Iso. b, *M* = 1
E=-3847.128704
C -0.800831 2.448046 -0.002768
O -0.634448 1.936576 1.121093
Cu -0.003405 0.001593 1.283072
O -1.360002 -1.518208 1.121015
C -1.720842 -1.916966 -0.002779
O -1.361240 -1.513641 -1.125338
Cu 0.004298 -0.001597 -1.282762
O -0.632321 1.934520 -1.125133
O 1.996117 -0.418173 -1.119344
C 2.520530 -0.531111 0.005183
O 1.989918 -0.421193 1.126881
H -1.147612 3.502023 -0.003871
H 3.606585 -0.758238 0.007704
H -2.462189 -2.742517 -0.004042

Cu2(HCO2)3-, Iso. a, *M* = 1
E=-3847.127996
Cu -1.621967 0.228853 0.007907
Cu 1.621971 0.228980 0.010913
O -2.240752 -1.530725 0.025290
C -3.501198 -1.728088 -0.006043
H -3.762447 -2.807718 0.007484
O -4.392799 -0.898894 -0.046862
O -1.122829 2.047153 -0.005325
C -0.000117 2.584318 -0.007303
H -0.000135 3.686689 -0.015249
O 1.122613 2.047220 -0.001512
O 2.240982 -1.530504 0.028459
C 3.501283 -1.728014 -0.007355
O 4.392806 -0.898971 -0.052599
H 3.762483 -2.807653 0.006598

Cu2(HCO2)3-, *M* = 3
E=-3847.033148
Cu -1.203873 -0.005609 -0.026209
Cu 1.203874 -0.005609 -0.026207
O 1.121491 2.011776 0.102263
C -0.000002 2.554835 0.130198
O -1.121494 2.011776 0.102258
H -0.000002 3.660983 0.185871
O 2.259619 -1.923733 0.224032
C 3.297998 -1.288256 -0.030278
O 3.312503 -0.059081 -0.262084
H 4.261142 -1.834500 -0.055530
O -2.259624 -1.923732 0.224051
C -3.297998 -1.288255 -0.030274
O -3.312496 -0.059082 -0.262097
H -4.261145 -1.834494 -0.055525

Cu2(HCO2)5-, Iso.1, *M* = 1
E=-4225.431002
C 0.000001 0.000001 2.522655
O -1.101443 0.201466 1.979438
Cu -1.522124 0.042291 0.058499
C -3.579145 -0.059352 -1.319664
O -2.382063 -0.104815 -1.722593
O 1.101443 -0.201464 1.979437
Cu 1.522124 -0.042291 0.058497
C 3.579148 0.059350 -1.319660
O 3.904499 -0.045814 -0.135895
O 1.084701 -1.961702 -0.237456
C -0.040759 -2.478497 -0.108762
O -1.135780 -1.919032 0.088305
O 1.135777 1.919032 0.088299
C 0.040756 2.478497 -0.108763
O -1.084705 1.961703 -0.237453
O 2.382068 0.104814 -1.722593
O -3.904499 0.045813 -0.135900
H 0.000002 0.000001 3.625098
H -0.072360 -3.579120 -0.178749
H 0.072357 3.579120 -0.178751
H -4.357905 -0.119819 -2.102022
H 4.357911 0.119816 -2.102016

Cu2(DCO2)5-, Iso.1, *M* = 1
E=-4225.447085
C 0.000001 0.000001 2.522655
O -1.101443 0.201466 1.979438
Cu -1.522124 0.042291 0.058499
C -3.579145 -0.059352 -1.319664
O -2.382063 -0.104815 -1.722593
O 1.101443 -0.201464 1.979437
Cu 1.522124 -0.042291 0.058497
C 3.579148 0.059350 -1.319660
O 3.904499 -0.045814 -0.135895
O 1.084701 -1.961702 -0.237456
C -0.040759 -2.478497 -0.108762
O -1.135780 -1.919032 0.088305
O 1.135777 1.919032 0.088299
C 0.040756 2.478497 -0.108763
O -1.084705 1.961703 -0.237453
O 2.382068 0.104814 -1.722593
O -3.904499 0.045813 -0.135900
H(Iso=2) 0.000002 0.000001 3.625098
H(Iso=2) -0.072360 -3.579120 -0.178749
H(Iso=2) 0.072357 3.579120 -0.178751
H(Iso=2) -4.357905 -0.119819 -2.102022
H(Iso=2) 4.357911 0.119816 -2.102016

Cu2(HCO2)5-, Iso.1, *M* = 3
E=-4225.430730
C -0.000001 0.000001 2.523660
O -1.102914 0.193080 1.980354
Cu -1.522235 0.040390 0.057939
C -3.581820 -0.057059 -1.317303
O -2.385403 -0.100720 -1.722429
O 1.102913 -0.193079 1.980355
Cu 1.522235 -0.040391 0.057940
C 3.581820 0.057059 -1.317300
O 3.905116 -0.043792 -0.132607
O 1.087088 -1.960789 -0.232317
C -0.038483 -2.478961 -0.110246
O -1.135207 -1.920355 0.079381
O 1.135207 1.920355 0.079380
C 0.038484 2.478960 -0.110248
O -1.087087 1.960788 -0.232320
O 2.385404 0.100718 -1.722428
O -3.905116 0.043795 -0.132610
H -0.000001 0.000001 3.626261
H -0.068409 -3.579775 -0.179583
H 0.068409 3.579774 -0.179586
H -4.361913 -0.114994 -2.098520
H 4.361915 0.114994 -2.098517

H2CO2

E=-189.716228
C -0.133157 0.396832 0.000034
O 1.106445 -0.088837 -0.000003
O -1.124308 -0.263699 -0.000008
H -0.107719 1.494450 -0.000088
H 1.049568 -1.055152 -0.000030

H2
E=-1.158059
H -0.000000 -0.000000 0.370754
H 0.000000 -0.000000 -0.370754

HCO2
E=-189.043195
C -0.000019 0.435518 -0.000238
O 1.027898 -0.259003 0.000055
O -1.027742 -0.259260 0.000055
H -0.001133 1.532993 0.000553

H
E=-0.498614
H 0.000000 0.000000 0.000000

TS12
E=-2018.122803
c -0.550491 -2.799368 0.000000
h -0.683347 -1.161005 0.000000
cu 0.088826 0.197500 -0.000000
o 1.154169 1.776333 -0.000000
c 0.635682 2.938560 -0.000000
o -0.543301 3.248302 -0.000000
h 1.402098 3.747572 -0.000000

TS7
E=-3658.517728
Cu 0.627292 0.034838 -0.271120
Cu -1.612696 -0.995035 0.147570
H -0.453565 -1.983961 0.032498
O 2.473630 -0.338061 -0.790398
C 3.372148 -0.082092 0.066235
H 4.400067 -0.320788 -0.294915
O 3.242425 0.376143 1.192321
O -0.715642 1.621517 -0.184805
C -1.939945 1.619763 0.012926
H -2.441255 2.605542 0.026025
O -2.690632 0.630263 0.200927

Cu2H(HCO2)2.CO2-
E=-3847.097388
H 2.068807 -0.884249 -0.002832
Cu -1.862554 0.078602 0.001443
Cu 1.344455 0.450299 -0.005171
C 4.679349 -1.188241 0.005708
O 4.733258 -1.149905 1.158003
O 4.721900 -1.245861 -1.146251
O 0.610359 2.242376 -0.008984
C -0.569110 2.629788 -0.007951
O -1.616114 1.951628 -0.004206
O -2.267597 -1.745440 0.007637
C -3.488317 -2.113668 0.007264
O -4.489160 -1.418397 0.003926
H -0.719226 3.723646 -0.010719
H -3.597412 -3.220016 0.010516

TS6
E=-3847.087285
H -1.992662 -1.073685 -0.001408
C -3.497321 -1.301354 0.000067
O -3.818470 -1.384745 1.141844
O -3.822296 -1.376936 -1.141185
Cu -1.398009 0.376702 0.000004
Cu 1.612895 0.169812 -0.000942
O 1.356379 2.044057 0.000337
C 0.282876 2.675492 0.001404
O -0.878252 2.226325 0.001656
H 0.374816 3.773800 0.002231
O 1.980486 -1.658749 -0.002528
C 3.207171 -2.013209 -0.000377
O 4.194456 -1.299707 0.002527
H 3.331378 -3.116544 -0.001415

Cu2(HCO2)3-.CO2.H2CO2, *M* = 3
E=-4225.359513
H -2.345257 0.558248 0.057849
O -2.747688 1.468797 0.228345
C -1.948731 2.453237 0.424283
H -2.501520 3.391147 0.566962
O -0.735700 2.465939 0.471521
Cu 0.800812 1.125960 0.334475
O 0.365579 0.630593 2.139132
O 1.158781 1.805302 -1.454576
O 2.589566 0.303040 0.377920
C 2.867146 -0.717705 -0.287938
O 2.091843 -1.481393 -0.883565
C 0.253152 1.369563 -2.230057
O -0.676899 0.640949 -1.903928
C 0.412124 -0.578624 2.531871
O 0.553926 -1.581424 1.850321
Cu 0.187505 -1.673953 -0.508840
O -1.672134 -2.177719 -0.502488
C -2.558411 -1.374696 -0.156986
O -3.746751 -1.451510 0.021010
H 0.299951 -0.687953 3.628243
H 0.347877 1.699969 -3.281834
H 3.941879 -0.960844 -0.351229

Cu2(HCO2)3-.CO2.H2CO2 *M* = 1
E=-4225.443576
H 2.331652 -1.410851 0.288985
O 3.151975 -1.983861 0.093300
C 4.274727 -1.343517 0.254191
H 5.132296 -1.997893 0.021321
O 4.419586 -0.203644 0.608532
Cu -2.163053 0.111672 0.822279
O -0.583905 -0.477174 2.173841
O -2.998356 -1.515029 0.003460
O -1.987081 2.094330 0.566978
C -1.125034 2.535261 -0.214912
O -0.366903 1.893968 -0.973111
C -2.472114 -2.054810 -0.988942
O -1.451981 -1.704744 -1.615085
C 0.548469 -0.743770 1.771756
O 0.968742 -0.703222 0.581024
Cu -0.400636 -0.111670 -1.036000
O 2.440217 0.879708 -1.455720
C 2.399253 1.544006 -0.511576
O 2.386513 2.241260 0.405621
H 1.294556 -1.058168 2.527706
H -2.977003 -2.963690 -1.366463
H -1.016804 3.634786 -0.247459

Cu2(HCO2)5-, Iso.2
E=-4225.423658
H 2.945645 1.239359 -0.550359
O 0.547018 1.764521 0.469235

C -0.350817 2.598968 0.529804
H -0.100283 3.647909 0.769817
O -1.583593 2.371571 0.337312
Cu -1.713876 0.475940 -0.054481
O -1.150693 0.739235 -1.945711
O -1.637842 -0.016454 1.867032
O -2.464017 -1.277951 -0.502070
C -1.649072 -2.217786 -0.613385
O -0.418363 -2.170717 -0.462630
C -0.662437 -0.558321 2.434249
O 0.407745 -0.950502 1.942474
C -0.026691 0.454648 -2.413668
O 0.926987 -0.118952 -1.860060
Cu 0.946940 -0.762857 0.030635
O 2.794400 -0.552887 0.356782
C 3.516053 0.425353 -0.060587
O 4.722350 0.508993 0.051749
H 0.135240 0.750489 -3.463838
H -0.777028 -0.710223 3.520824
H -2.076562 -3.198971 -0.876307

TS9
E=-2207.253190
C -2.632383 -0.771319 0.117312
H -1.671237 0.456598 -0.805247
O -3.720005 -0.471557 -0.169331
O -1.770796 -1.392616 0.637110
Cu -0.198359 0.030617 -0.318030
O 1.196765 -1.186738 0.050502
C 2.461674 -0.964492 0.028178
O 3.331454 -1.795152 0.190807
O 0.198126 2.095250 1.216317
C 0.725204 2.393562 0.151746
O 0.783672 1.654548 -0.879116
H 1.205488 3.385574 0.035164
H 2.737455 0.093524 -0.160781

TS10
E=-2018.648412
H 1.618679 -1.101732 -0.000003
O 2.882634 -0.897585 -0.000001
Cu 0.053627 -0.255614 -0.000001
O 2.107584 1.203518 0.000004
C 3.000258 0.375929 0.000002
H 4.059325 0.709526 0.000003
O -1.737397 0.465735 0.000000
C -2.819888 -0.203368 -0.000001
O -3.956465 0.238653 -0.000000
H -2.686248 -1.312913 -0.000003

TS11
E=-2018.654846
C -2.727729 -0.380014 0.021781
O -3.918578 -0.262350 -0.147735
O -1.806080 0.462534 -0.200558
Cu 0.028426 0.288496 0.066985
O 1.858179 0.279268 0.352223
C 2.753118 -0.378495 -0.284287
O 3.949937 -0.343074 -0.082729
H -1.672133 -2.470424 0.910865

TS1 *M*=1
E=-4225.386313
h 2.178865 -0.116670 -1.275642
cu 1.390156 0.047487 0.121053
c 3.849723 -0.077417 -0.943418
o 3.930433 0.064469 0.229056
o 4.290406 -0.208270 -2.021594
cu -1.611892 -0.042314 -0.016276
o -1.208823 1.919339 -0.081372
c -0.106552 2.486227 -0.198401
o 1.031702 1.979984 -0.214128
o 0.879804 0.275960 2.038848
c -0.251696 0.118281 2.527795
o -1.326626 -0.087768 1.931190
o 1.065007 -1.922673 0.246781
c -0.025079 -2.489030 0.037095
o -1.139317 -1.975810 -0.174180
o -2.351436 -0.006096 -1.859343
c -3.571070 -0.031118 -1.528686
o -3.966249 -0.065666 -0.361862
h -0.317870 0.165789 3.627642
h -0.142396 3.584147 -0.302756
h 0.002090 -3.592115 0.036970
h -4.301498 -0.020575 -2.358208

TS1 *M*=3
E= -4225.385977
h -2.185110 0.107795 -1.276467
cu -1.390687 -0.044137 0.118553
c -3.855193 0.071204 -0.938405
o -3.931671 -0.061320 0.235402
o -4.299405 0.193498 -2.016125
cu 1.613131 0.039816 -0.015365
o 1.208001 -1.920905 -0.080742
c 0.104635 -2.486963 -0.190982
o -1.032912 -1.979026 -0.202885
o -0.881497 -0.256566 2.038844
c 0.250779 -0.107665 2.528861
o 1.328449 0.085460 1.932932
o -1.062881 1.926354 0.223811
c 0.030052 2.490312 0.022937
o 1.145837 1.974360 -0.172914
o 2.353043 0.002725 -1.858304
c 3.572660 0.025656 -1.527322
o 3.967485 0.059303 -0.360378
h 0.314832 -0.151088 3.629155
h 0.138581 -3.585365 -0.292312
h 0.004333 3.593548 0.016131
h 4.303275 0.014079 -2.356648

$Cu_2(HCO_2)_4H^-$ Iso.1 *M*=1
E= -4036.823144
H 2.435692 -0.013994 -2.146042
Cu 1.900941 0.058770 -0.695494
Cu -0.994999 -0.061933 0.193192
O -0.621359 1.894284 0.299604
C 0.356497 2.489669 -0.195376
O 1.366531 2.009764 -0.738478
O 2.009032 0.184113 1.337181
O 1.515832 -1.930013 -0.508617
C 1.121579 -0.127082 2.146862
C 0.404612 -2.484933 -0.455608
O -0.719979 -1.963439 -0.316412
O -0.080802 -0.367470 1.915628
H 0.408681 -3.585875 -0.545372
H 1.424313 -0.202937 3.206019
O -2.277391 0.217350 -1.293490
C -3.352046 0.141239 -0.631208
O -3.411077 -0.053865 0.582285
H -4.284625 0.263126 -1.214451
H 0.313471 3.592251 -0.143022

$Cu_2(HCO_2)_4H^-$ Iso.1 *M*=3
E= -4036.822922
H 2.457908 -0.013273 -2.137239

Cu 1.905718 0.056922 -0.692949
Cu -0.995972 -0.060292 0.192486
O -0.621973 1.895072 0.290577
C 0.358441 2.489217 -0.200783
O 1.370966 2.007461 -0.737433
O 2.008910 0.178915 1.341054
O 1.511501 -1.930640 -0.514515
C 1.118863 -0.126338 2.150246
C 0.400205 -2.484878 -0.457734
O -0.723164 -1.962703 -0.311389
O -0.084294 -0.361447 1.917941
H 0.402776 -3.585673 -0.550818
H 1.419949 -0.201400 3.210059
O -2.279590 0.214163 -1.294843
C -3.353682 0.141000 -0.631342
O -3.411705 -0.049434 0.582962
H -4.286768 0.260944 -1.214187
H 0.315344 3.592058 -0.151542

TS2 *M*=1
E= -4036.813310
h -0.514121 0.000000 -1.711008
cu -1.628579 0.000000 -0.572480
o 2.719138 0.000000 -0.850136
cu 0.891482 0.000000 -0.171665
o -1.562258 -2.007697 -0.534216
c -0.434674 -2.530597 -0.406322
o 0.668427 -1.975510 -0.242210
o -1.562258 2.007697 -0.534216
c -0.434674 2.530597 -0.406322
o 0.668427 1.975510 -0.242210
c -1.511314 0.000000 1.998141
o -2.512443 0.000000 1.245736
o -0.318478 0.000000 1.644605
h -1.710499 0.000000 3.084220
c 3.613092 0.000000 0.055387
o 3.447211 0.000000 1.262938
h 4.647248 0.000000 -0.352943
h -0.411760 -3.633518 -0.443849
h -0.411760 3.633518 -0.443849

TS2 *M*=3
E= -4036.810445
H -0.514438 0.000000 -1.699865
Cu -1.625584 0.000000 -0.520524
O 2.747131 0.000000 -0.848048
Cu 0.891303 0.000000 -0.300608
O -1.556151 -2.003442 -0.490880
C -0.426933 -2.534197 -0.409098
O 0.684255 -1.983557 -0.296250
O -1.556151 2.003442 -0.490880
C -0.426933 2.534197 -0.409098
O 0.684255 1.983557 -0.296250
C -1.539025 0.000000 2.007957
O -2.555180 0.000000 1.270318
O -0.357687 0.000000 1.623355
H -1.720527 0.000000 3.097532
C 3.627364 0.000000 0.074019
O 3.448964 0.000000 1.277377
H 4.665205 0.000000 -0.325360
H -0.413831 -3.637885 -0.442048
H -0.413831 3.637885 -0.442048

Cu2(HCO2)4H- Iso.2 *M*=1
E= -4036.829210
h 0.179840 -0.000012 -1.250796
cu -1.331459 -0.000011 -0.586401
o 3.533536 -0.000008 -0.812433
cu 1.268131 0.000000 -0.015252
o -1.191594 -1.982111 -0.502591
c -0.121750 -2.513966 -0.140979
o 0.953221 -1.967494 0.173110
o -1.191610 1.982089 -0.502613
c -0.121768 2.513956 -0.141011
o 0.953207 1.967495 0.173084
c -3.384515 0.000004 0.970103
o -3.274098 -0.000016 -0.293934
o -2.454181 0.000049 1.767525
h -4.427565 0.000005 1.345170
c 3.721423 0.000020 0.406900
o 2.800236 0.000021 1.269105
h 4.756298 0.000030 0.799049
h -0.131058 -3.616234 -0.092778
h -0.131083 3.616225 -0.092824

Cu2(HCO2)4H- Iso.2 *M*=3
E= -4036.810445
H -0.514438 0.000000 -1.699865
Cu -1.625584 0.000000 -0.520524
O 2.747131 0.000000 -0.848048
Cu 0.891303 0.000000 -0.300608
O -1.556151 -2.003442 -0.490880
C -0.426933 -2.534197 -0.409098
O 0.684255 -1.983557 -0.296250
O -1.556151 2.003442 -0.490880
C -0.426933 2.534197 -0.409098
O 0.684255 1.983557 -0.296250
C -1.539025 0.000000 2.007957
O -2.555180 0.000000 1.270318
O -0.357687 0.000000 1.623355
H -1.720527 0.000000 3.097532
C 3.627364 0.000000 0.074019
O 3.448964 0.000000 1.277377
H 4.665205 0.000000 -0.325360
H -0.413831 -3.637885 -0.442048
H -0.413831 3.637885 -0.442048

TS3
E= -4036.794537
H -0.772394 0.000000 0.822850
Cu 0.915674 0.000000 0.639461
O -2.064643 0.000000 1.877760
Cu -1.224836 0.000000 -0.837961
O 0.773966 -1.932301 0.518232
C 0.101901 -2.456245 -0.399036
O -0.687384 -1.907062 -1.183439
O 0.773966 1.932301 0.518232
C 0.101901 2.456245 -0.399036
O -0.687384 1.907062 -1.183439
C 3.442973 0.000000 -0.100958
O 2.873961 0.000000 1.029548
O 2.887115 0.000000 -1.194712
H 4.553237 0.000000 -0.060368
C -3.099239 0.000000 1.181375
O -3.183638 0.000000 -0.059718
H -4.056754 0.000000 1.742571
H 0.234372 -3.545366 -0.511151
H 0.234372 3.545366 -0.511151

Cartesian coordinates of optimized ions and molecules (in Å, calculated at the B3LYP /def2TZVP level) along with electronic energies (in Hartree) including zero point energy

CuH(CO2)-
E=-1829.847146
C 1.234830 1.102175 0.000000
O 1.829425 2.160882 0.000000
O 0.000000 0.818835 0.000000
Cu -0.713399 -0.969638 0.000000
H -1.355800 -2.331271 0.000000

Cu(HCO2)-
E=-1829.849931
C -1.590648 0.282867 0.000275
O -2.781377 -0.024368 -0.000378
O -0.588562 -0.510561 0.000367
Cu 1.304674 0.041761 -0.000080
H -1.332150 1.371160 0.000769

Cu(HCOO)H-
E=-1830.482989
Cu -0.861009 -0.697150 -0.000000
O 2.206953 0.500126 0.000000
C 1.252725 1.272402 0.000000
O 0.000000 1.023048 0.000000
H 1.463327 2.372279 0.000000

Cu(HCOO)2-
E=-2019.175428
C -2.774974 -0.323672 0.000161
O -3.026758 0.874543 0.000263
O -1.635646 -0.914947 -0.000086
Cu -0.000014 -0.000151 -0.000173
O 1.635446 0.914870 -0.000094
C 2.774920 0.323973 0.000146
O 3.027078 -0.874205 0.000264
H -3.618739 -1.052843 0.000155
H 3.618513 1.053326 0.000242

Cu(HCOO)3-
E=-2208.394944
Cu 0.000103 0.132181 -0.000071
O 1.154089 -1.145020 0.882956
C 2.219133 -1.375577 0.206108
O 2.538808 -0.896072 -0.875636
H 2.894585 -2.101897 0.709062
O 0.796550 1.917599 0.757420
C 0.001287 2.528980 0.000193
O -0.794435 1.918237 -0.757115
H 0.001718 3.632081 0.000172
O -1.155348 -1.143711 -0.883035
C -2.220565 -1.373533 -0.206206
O -2.539898 -0.893958 0.875603
H -2.896557 -2.099269 -0.709282

Cu(DCOO)3-
E= -2208.404239
Cu 0.000103 0.132181 -0.000071
O 1.154090 -1.145019 0.882956
C 2.219135 -1.375575 0.206108
O 2.538809 -0.896069 -0.875636
H(Iso=2) 2.894587 -2.101894 0.709062
O 0.796548 1.917600 0.757420
C 0.001284 2.528980 0.000193
O -0.794437 1.918236 -0.757115
H(Iso=2) 0.001714 3.632081 0.000172
O -1.155347 -1.143712 -0.883035
C -2.220563 -1.373535 -0.206206
O -2.539897 -0.893960 0.875603
H(Iso=2) -2.896555 -2.099272 -0.709282

Cu(HCO2)CO2-
E=-2018.536985
C 2.683373 -0.189777 -0.000157
O 1.673275 0.588637 -0.000666
Cu -0.064437 -0.110839 -0.000124
O -1.767395 -0.885797 0.000263
C -2.839256 -0.176187 0.000612
O -2.957365 1.041384 0.000767
O 3.871327 0.033429 -0.000356
H -3.754770 -0.811116 0.000784

Cu(HCOO)2H-
E=-2019.712589
C 2.882763 0.217648 0.000193
O 4.009629 -0.249555 0.000498
O 1.781398 -0.459158 -0.000507
Cu 0.000002 0.151178 -0.000336
O -1.781397 -0.459175 -0.000073
C -2.882767 0.217646 0.000457
O -4.009632 -0.249546 0.000722
H 2.740396 1.319166 0.000594
H -2.740365 1.319156 0.000649
H -0.000037 1.705211 -0.000519

TS12
E=-2019.142816
H 1.298625 -0.029541 0.118568
Cu -0.222348 0.050357 -0.214531
C 2.846496 -0.037826 0.159858
O 3.162807 -1.163501 -0.071677
O 3.162880 1.081939 0.418366
O -2.025592 0.191707 -0.800763
C -3.022832 0.004372 -0.014957
O -3.024380 -0.280228 1.175887
H -3.998258 0.130572 -0.541077

TS9
E=-2208.354826
cu -0.138504 -0.043046 -0.211806
h -1.638653 0.335149 -0.510378
c -2.681619 -0.860000 0.065676
o -1.960806 -1.663835 0.570856
o 1.318865 -1.175154 0.166718
c 2.579789 -0.953053 -0.031083
o 3.481414 -1.753670 0.146859
o -3.731886 -0.435627 -0.261232
h 2.807143 0.070737 -0.394244
o 0.645044 1.622797 -0.866250
c 0.579341 2.564310 0.007343
h 1.011275 3.516393 -0.370054
o 0.118846 2.507805 1.138730

TS10
E=-2019.676819
H -1.366439 1.269385 -0.000000
O -2.704479 1.335430 -0.000000
Cu 0.000000 0.271309 0.000000
O -2.354819 -0.894974 -0.000000
C -3.065845 0.101628 -0.000000

H -4.172385 -0.014735 -0.000000
O 1.657898 -0.722153 0.000000
C 2.845199 -0.237582 0.000000

TS11
E=-2019.678600
c -2.645015 -0.493725 -0.000000
o -3.857466 -0.556124 -0.000000
o -1.878687 0.524802 -0.000000
cu -0.000000 0.364373 -0.000000
o 1.852299 0.473000 0.000000
c 2.704530 -0.496534 0.000000
o 3.920470 -0.381833 0.000000
h -0.897844 -2.084297 -0.000000

TS8
E=-2208.356201
O 2.652082 1.388988 0.348529
H 2.366127 0.052538 0.190233
O 3.110854 -1.915881 0.129621
C 2.133576 -1.198469 0.054048
O 0.907659 -1.427318 -0.135615
C 1.621368 2.131613 0.200537
H 1.842346 3.211028 0.349720
O 0.468076 1.803326 -0.077112
Cu -0.544853 -0.204992 -0.284553
O -2.337171 0.311121 -0.719339
C -3.270394 0.012662 0.106840
O -3.182034 -0.579352 1.176836
H -4.270785 0.359274 -0.239827

CO2
E=-188.658232
O 0.000000 0.000000 1.159737
C 0.000000 0.000000 0.000000
O 0.000000 0.000000 -1.159737

Cu(HCO2)2
E=-2019.059259
C 2.321774 -0.000422 0.000045
O 1.683079 1.089093 0.000040
O 1.682413 -1.089466 -0.000010
Cu 0.000016 0.000454 -0.000025
O -1.683195 1.089093 -0.000045
C -2.321768 -0.000424 0.000025
O -1.682359 -1.089531 0.000039
H 3.416889 -0.000720 0.000081
H -3.416882 -0.000890 0.000053

Cu(DCO2)2
E= -2019.065671
C 2.321774 -0.000422 0.000045
O 1.683079 1.089093 0.000040
O 1.682413 -1.089466 -0.000010
Cu 0.000016 0.000454 -0.000025
O -1.683195 1.089093 -0.000045
C -2.321768 -0.000424 0.000025
O -1.682359 -1.089531 0.000039
H(Iso=2) 3.416889 -0.000720 0.000081
H(Iso=2) -3.416882 -0.000890 0.000053

HCOOH
E=-189.814996
C -0.136495 0.397716 0.000127
O 1.116708 -0.088742 -0.000152
O -1.133546 -0.264034 -0.000069
H -0.104214 1.494705 0.000201
H 1.057889 -1.058794 0.000811

H2
E=-1.169577
H 0.000000 -0.000000 0.371997
H 0.000000 -0.000000 -0.371997

H
E=-0.502154
H 0.000000 0.000000 0.000000

TS4
E= -4227.496345
Cu 1.449655 -0.128511 0.087787
Cu -1.456643 0.539919 0.022289
O -1.427316 -0.791892 1.478782
C -0.463277 -1.542957 1.757238
O 0.705337 -1.536437 1.303491
O 1.333269 1.236042 1.519422
C 0.348738 1.961530 1.783325
O -0.801775 1.926696 1.288206
O -0.793798 1.662264 -1.510933
C 0.409553 1.823644 -1.820256
O 1.435936 1.300296 -1.326856
O 1.996664 -1.474715 -1.220306
C 3.251166 -1.583389 -0.994667
O 3.878524 -0.952227 -0.143032
H 3.769171 -2.316440 -1.643308
H -0.676932 -2.316932 2.512605
H 0.592941 2.530587 -2.648394
H 0.515494 2.733729 2.552693
O -2.685106 -0.264311 -1.190577
C -3.491605 -1.271807 -1.089251
O -3.672601 -2.042812 -0.167486
H -4.078520 -1.387135 -2.029806

Cu2(HCO2)2H- Iso.1
E=-3660.383812
H -4.816655 1.093879 0.000943
Cu -1.252339 -0.120435 -0.000702
C -3.979178 0.360406 0.000701
O -4.234016 -0.837378 0.001629
Cu 1.252337 -0.120377 -0.000194
O 2.829520 0.933661 0.000820
C 3.979187 0.360369 0.000775
O 4.233983 -0.837422 -0.000005
O -2.829493 0.933655 -0.000599
H 4.816688 1.093815 0.001572
H 0.000003 -1.068935 -0.000143

Cu2(HCO2)2H- Iso.2
E=-3660.367725
O 3.957577 0.280266 0.001067
H 4.032562 -1.735961 0.001297
C 3.408699 -0.814316 0.000870
O 2.152665 -1.086539 0.000088
Cu 0.891393 0.293333 -0.000725
O -0.264678 1.781984 -0.001701
C -1.519505 1.856857 0.000420
H -1.918716 2.886984 0.000225
O -2.369659 0.943565 0.002739
Cu -2.234625 -0.992321 -0.000116
H -2.302527 -2.489812 -0.002390

Cu2(HCO2)3- Iso. 1
E=-3849.049192
C -0.808469 2.458663 0.000225
O -0.640601 1.944096 1.131976
Cu -0.004141 -0.000145 1.268450

O -1.364916 -1.530195 1.127378
C -1.726957 -1.928432 -0.005440
O -1.367041 -1.521529 -1.135890
Cu 0.004384 -0.000292 -1.267877
O -0.638714 1.946194 -1.131849
O 2.008815 -0.415283 -1.128494
C 2.534722 -0.529651 0.004649
O 2.002478 -0.422345 1.135222
H -1.156299 3.512387 0.001252
H 3.621068 -0.756089 0.006714
H -2.467738 -2.754598 -0.007932

Cu2(HCO2)3- Iso. 2
E=-3849.053933
O -4.459407 -0.901777 -0.000403
C -3.555439 -1.726984 -0.000381
H -3.803658 -2.810657 -0.000595
O -2.284670 -1.525021 0.000276
Cu -1.652237 0.230435 0.000244
O -1.132654 2.041616 0.000053
C -0.000008 2.577173 -0.000161
H -0.000014 3.679364 -0.000262
O 1.132644 2.041629 -0.000351
Cu 1.652251 0.230454 0.000041
O 2.284656 -1.525011 0.000231
C 3.555422 -1.726997 -0.000094
H 3.803621 -2.810674 -0.000668
O 4.459406 -0.901807 -0.000170

Cu2(HCOO)5- Iso.1 *M*=3
E=-4227.503618
C 0.000000 -0.000006 2.569445
O 1.128313 -0.001336 2.026634
Cu 1.500425 -0.000370 0.084415
C 3.564021 0.001015 -1.439851
O 2.310135 0.000638 -1.694731
O -1.128311 0.001310 2.026631
Cu -1.500424 0.000323 0.084412
C -3.564024 -0.000996 -1.439848
O -4.064112 -0.000629 -0.314760
O -1.133896 1.969050 -0.034595
C -0.000531 2.502225 -0.074844
O 1.132385 1.968262 -0.033545
O -1.132384 -1.968302 -0.033532
C 0.000531 -2.502266 -0.074824
O 1.133896 -1.969090 -0.034570
O -2.310139 -0.000692 -1.694733
O 4.064113 0.000976 -0.314764
H -0.000001 -0.000007 3.672438
H -0.000132 3.603515 -0.156880
H 0.000133 -3.603558 -0.156832
H 4.210176 0.001596 -2.339730
H -4.210183 -0.001502 -2.339725

Cu2(HCOO)5- Iso1. *M*=1
E=-4227.504360
C 0.000247 0.000118 2.569835
O -1.128405 -0.008879 2.028099
Cu -1.499213 -0.001999 0.087173
C -3.551174 0.002523 -1.452614
O -2.295441 0.004217 -1.697733
O 1.128606 0.008996 2.027461
Cu 1.499265 0.001990 0.086565
C 3.551858 -0.002571 -1.451944
O 4.060198 0.002081 -0.330468
O 1.134782 -1.969350 -0.020438
C 0.001994 -2.503030 -0.067019
O -1.132347 -1.971650 -0.033815
O 1.131652 1.971513 -0.035173
C -0.002662 2.503008 -0.067249
O -1.135461 1.969456 -0.019335
O 2.296330 -0.004338 -1.698036
O -4.060471 -0.002056 -0.331594
H 0.000573 0.000129 3.672518
H 0.003563 -3.604234 -0.147274
H -0.004212 3.604203 -0.147615
H -4.190306 0.005531 -2.357530
H 4.191756 -0.005606 -2.356308

Cu2(DCOO)5- Iso.1 *M*=1
E=-4227.520127
C -0.000247 -0.000118 2.569835
O 1.128405 0.008879 2.028099
Cu 1.499213 0.001999 0.087173
C 3.551174 -0.002523 -1.452614
O 2.295441 -0.004217 -1.697733
O -1.128606 -0.008996 2.027461
Cu -1.499265 -0.001990 0.086565
C -3.551858 0.002571 -1.451944
O -4.060198 -0.002081 -0.330468
O -1.134782 1.969350 -0.020438
C -0.001994 2.503030 -0.067019
O 1.132347 1.971650 -0.033815
O -1.131652 -1.971513 -0.035173
C 0.002662 -2.503008 -0.067249
O 1.135461 -1.969456 -0.019335
O -2.296330 0.004338 -1.698036
O 4.060471 0.002056 -0.331594
H(Iso=2) -0.000573 -0.000129 3.672518
H(Iso=2) -0.003563 3.604234 -0.147274
H(Iso=2) 0.004212 -3.604203 -0.147615
H(Iso=2) 4.190306 -0.005531 -2.357530
H(Iso=2) -4.191756 0.005606 -2.356308

Cu2(HCOO)5- Iso2. *M*=1
E= -4227.497680
H 2.575789 1.618825 -0.197639
O 0.180926 2.612864 0.167088
C -0.989019 2.967511 0.174095
H -1.244226 4.045377 0.243226
O -2.031698 2.218111 0.108446
Cu -1.665229 0.335447 -0.024327
O -1.233361 0.456452 -1.966570
O -1.454642 0.137858 1.944024
O -2.192003 -1.567528 -0.211503
C -1.385375 -2.521913 -0.299780
O -0.136016 -2.486286 -0.259842
C -0.427941 -0.338532 2.489343
O 0.610512 -0.799111 1.966150
C -0.113665 0.166459 -2.454479
O 0.890577 -0.335451 -1.900536
Cu 1.063142 -0.917425 0.010034
O 2.839251 -0.345134 0.228008
C 3.330255 0.841664 0.021074
O 4.517953 1.109921 0.069088
H -0.001753 0.382392 -3.530724
H -0.450715 -0.353890 3.592626
H -1.836107 -3.520048 -0.429340

TS7
E=-3660.359772
Cu 0.601342 0.002437 -0.262346
Cu -1.602173 -0.991654 0.140701
H -0.428458 -1.933172 0.014069
O 2.455596 -0.260681 -0.802970
C 3.396619 -0.044295 0.033547

H 4.407496 -0.231897 -0.403525
O 3.322895 0.326359 1.202178
O -0.721456 1.649725 -0.170159
C -1.952666 1.630591 0.027479
H -2.468321 2.609515 0.048587
O -2.700828 0.625230 0.208754

TS6
E=-3849.017736
H -1.916884 -1.171784 -0.137533
Cu 1.568106 0.202671 0.017484
Cu -1.423810 0.336885 -0.081186
C -3.273038 -1.342849 0.058987
O -3.627546 -2.128377 -0.783418
O -3.681096 -0.716680 1.016109
O -0.970839 2.198126 -0.096194
C 0.187649 2.676221 -0.078573
O 1.284514 2.071025 -0.036202
O 1.970380 -1.618275 0.093920
C 3.211884 -1.958955 0.073926
O 4.196968 -1.235358 0.010722
H 0.251981 3.775572 -0.103248
H 3.342290 -3.061099 0.122610

Cu2(HCOO)2H(CO2)-
E=-3849.030070
H 2.179765 -0.753181 -0.000478
Cu -1.928357 0.065090 -0.000102
Cu 1.384585 0.520348 -0.000191
C 4.831654 -1.217304 0.000170
O 4.876149 -1.227871 1.159775
O 4.875882 -1.230003 -1.159421
O 0.552037 2.267840 0.000160
C -0.642624 2.631276 -0.000015
O -1.688559 1.935235 -0.000238
O -2.306544 -1.765243 0.000094
C -3.527868 -2.166501 0.000340
O -4.554473 -1.499305 0.000303
H -0.813620 3.722265 0.000021
H -3.599648 -3.276852 0.000615

TS5 *M*=3
E=-4227.453906
H 2.532246 0.447270 -0.031866
O 2.547648 1.730576 -0.137360
C 1.623374 2.517905 0.226345
H 1.956070 3.571437 0.282510
O 0.443419 2.295884 0.529218
Cu -1.076938 1.038426 0.093888
O -0.852099 1.495437 -1.793995
O -1.422994 0.876383 2.009960
O -2.714826 -0.006735 -0.315055
C -2.726391 -1.266144 -0.388185
O -1.776895 -2.065114 -0.292001
C -0.748722 -0.026651 2.599890
O 0.027330 -0.841820 2.092027
C -0.244391 0.638277 -2.512038
O 0.243524 -0.437947 -2.156274
Cu 0.139040 -1.491402 -0.116849
O 2.102288 -1.733617 -0.015132
C 2.973607 -0.853249 0.063608
O 4.181711 -0.827232 0.193723
H -0.159524 0.928627 -3.577175
H -0.901181 -0.054664 3.696407
H -3.726277 -1.703705 -0.562603

TS5 *M*=1
E=-4227.454495
H 2.529493 0.433603 -0.044311
O 2.543465 1.702541 -0.179093
C 1.652897 2.482479 0.275584
H 2.000097 3.528632 0.356546
O 0.491828 2.254243 0.641052
Cu -1.051124 1.049946 0.124034
O -0.766037 1.569286 -1.738070
O -1.443380 0.829526 2.023670
O -2.701799 0.049761 -0.361120
C -2.736470 -1.206192 -0.470204
O -1.805463 -2.027300 -0.375201
C -0.802292 -0.107800 2.597191
O -0.035352 -0.924963 2.079317
C -0.187344 0.716404 -2.485095
O 0.247397 -0.394776 -2.171284
Cu 0.115453 -1.492005 -0.155708
O 2.073250 -1.744769 -0.022615
C 2.955601 -0.878107 0.079989
O 4.159119 -0.869278 0.246789
H -0.075482 1.048231 -3.535438
H -0.978112 -0.166484 3.689032
H -3.740127 -1.619135 -0.679628

Cu2(HCOO)5- Iso.2 *M*=1
E=-4227.551636
H 2.044225 -1.831970 0.228945
O 2.866979 -2.429268 0.015898
C 4.022839 -1.851882 0.239251
H 4.849398 -2.542635 -0.008168
O 4.227708 -0.735200 0.656058
Cu -2.191830 0.194394 0.824413
O -0.872394 -0.934567 2.072572
O -3.269000 -1.076775 -0.289596
O -1.615436 2.111581 0.975619
C -0.636396 2.516475 0.309064
O 0.013950 1.904400 -0.579865
C -2.778887 -1.541376 -1.345153
O -1.679296 -1.265622 -1.890122
C 0.227596 -1.324719 1.649052
O 0.720748 -1.144553 0.495317
Cu -0.498375 0.068804 -1.069131
O 2.974044 1.217595 -1.508631
C 2.977578 1.766333 -0.486999
O 3.016987 2.352683 0.512851
H 0.858923 -1.900407 2.355677
H -3.393092 -2.293458 -1.873073
H -0.294219 3.544535 0.521359

Cu2(HCOO)3-+CO2+HCOOH *M*=1
E= -4227.551636
H 2.044225 -1.831970 0.228945
O 2.866979 -2.429268 0.015898
C 4.022839 -1.851882 0.239251
H 4.849398 -2.542635 -0.008168
O 4.227708 -0.735200 0.656058
Cu -2.191830 0.194394 0.824413
O -0.872394 -0.934567 2.072572
O -3.269000 -1.076775 -0.289596
O -1.615436 2.111581 0.975619
C -0.636396 2.516475 0.309064
O 0.013950 1.904400 -0.579865
C -2.778887 -1.541376 -1.345153
O -1.679296 -1.265622 -1.890122
C 0.227596 -1.324719 1.649052
O 0.720748 -1.144553 0.495317
Cu -0.498375 0.068804 -1.069131
O 2.974044 1.217595 -1.508631
C 2.977578 1.766333 -0.486999

O 3.016987 2.352683 0.512851
H 0.858923 -1.900407 2.355677
H -3.393092 -2.293458 -1.873073
H -0.294219 3.544535 0.521359

Cu2(HCO24)H- Iso.1 *M*=1
E= -4038.821602
H 2.191396 -0.000057 -2.236739
Cu 1.847492 0.000075 -0.741381
Cu -0.948734 -0.000156 0.254859
O -0.684759 1.978547 0.076305
C 0.369434 2.523744 -0.331013
O 1.446306 2.002909 -0.697465
O 2.120154 0.000225 1.299299
O 1.445795 -2.002658 -0.697161
C 1.222490 -0.000602 2.164084
C 0.368640 -2.523824 -0.331995
O -0.685966 -1.978979 0.074704
O -0.023909 -0.001219 1.999709
H 0.335579 -3.628391 -0.371081
H 1.551865 -0.000837 3.218913
O -2.212892 0.000647 -1.237154
C -3.376878 0.000891 -0.705113
O -3.626820 0.000624 0.498797
H -4.204389 0.001316 -1.444568
H 0.336201 3.628326 -0.369417

Cu2(HCO2)4H- Iso.1 *M*=3
E= -4038.820933
H -2.263729 -0.000027 -2.217858
Cu -1.861097 -0.000134 -0.736696
Cu 0.949049 0.000123 0.256226
O 0.688802 -1.976265 0.081170
C -0.360986 -2.520980 -0.339083
O -1.435439 -1.997999 -0.708738
O -2.112882 -0.000375 1.308940
O -1.435941 1.997841 -0.708325
C -1.214462 0.000145 2.173304
C -0.361298 2.520973 -0.339439
O 0.688841 1.976408 0.080133
O 0.031611 0.000591 2.007372
H -0.325174 3.625403 -0.387474
H -1.543122 0.000278 3.228615
O 2.207108 -0.000300 -1.240825
C 3.373636 -0.000165 -0.713804
O 3.628820 0.000181 0.488815
H 4.197545 -0.000376 -1.457194
H -0.324834 -3.625430 -0.386659

TS1 *M*=1
E= -4227.473847
h -2.379096 -0.738721 -0.000005
cu -1.318071 0.463681 -0.000001
c -3.823969 -1.231714 -0.000001
o -4.518026 -0.261970 0.000005
o -3.696818 -2.415751 -0.000005
cu 1.566139 -0.062325 0.000001
o 1.139663 -0.092012 1.977177
c 0.014903 0.057050 2.502772
o -1.096558 0.277084 1.958632
o -0.551476 2.348851 0.000003
c 0.657873 2.659493 0.000000
o 1.658133 1.900267 -0.000003
o -1.096554 0.277092 -1.958635
c 0.014907 0.057050 -2.502773
o 1.139665 -0.092021 -1.977176
o 1.905109 -1.988133 0.000005
c 3.183549 -2.025512 0.000002
o 3.919493 -1.036827 -0.000001
h 0.887182 3.738792 0.000000
h -0.010328 -0.012493 3.603769
h -0.010322 -0.012493 -3.603769
h 3.613969 -3.045247 0.000003

TS1 *M*=3
E= -4227.473148
h 2.399082 -0.724005 0.000003
cu 1.319402 0.462281 0.000001
c 3.842631 -1.222713 0.000000
o 4.538656 -0.254560 0.000002
o 3.711486 -2.406243 -0.000003
cu -1.568031 -0.062870 -0.000000
o -1.141432 -0.094497 -1.974426
c -0.016313 0.048564 -2.501441
o 1.095070 0.267979 -1.957048
o 0.549162 2.348000 0.000000
c -0.659830 2.660423 -0.000001
o -1.660437 1.901484 -0.000002
o 1.095068 0.267979 1.957051
c -0.016316 0.048565 2.501443
o -1.141435 -0.094495 1.974427
o -1.918361 -1.986470 -0.000001
c -3.197137 -2.017809 -0.000002
o -3.928297 -1.025792 -0.000003
h -0.888239 3.740168 -0.000001
h 0.008690 -0.026355 -3.602326
h 0.008686 -0.026354 3.602327
h -3.632034 -3.035631 -0.000003

TS2 *M*=1
E= -4038.813479
h -0.616669 0.000000 -1.741669
cu -1.646127 0.000000 -0.585180
o 2.656714 0.000000 -0.890784
cu 0.855376 0.000000 -0.088790
o -1.591350 -2.024136 -0.607654
c -0.457663 -2.535558 -0.435313
o 0.643714 -1.974421 -0.226881
o -1.591350 2.024136 -0.607654
c -0.457663 2.535558 -0.435313
o 0.643714 1.974421 -0.226881
c -1.462052 0.000000 2.069313
o -2.444840 0.000000 1.282274
o -0.246350 0.000000 1.774905
h -1.705770 0.000000 3.147705
c 3.656413 0.000000 -0.089297
o 3.654145 0.000000 1.135251
h 4.630160 0.000000 -0.632384
h -0.422662 -3.638374 -0.472736
h -0.422662 3.638374 -0.472736

TS2 *M*=3
E= -4038.806475
H -0.585484 0.000000 -1.681086
Cu -1.641568 0.000000 -0.483609
O 2.710242 0.000000 -0.942289
Cu 0.857109 0.000000 -0.358746
O -1.563029 -2.020545 -0.494119
C -0.422517 -2.544533 -0.429680
O 0.698231 -1.987889 -0.346503
O -1.563029 2.020545 -0.494119
C -0.422517 2.544533 -0.429680
O 0.698231 1.987889 -0.346503
C -1.543348 0.000000 2.087073
O -2.555443 0.000000 1.326397
O -0.348230 0.000000 1.731874

H	-1.756022	0.000000	3.173241
C	3.662808	0.000000	-0.078685
O	3.594708	0.000000	1.139572
H	4.660421	0.000000	-0.575877
H	-0.402742	-3.649167	-0.450543
H	-0.402742	3.649167	-0.450543

Cu2(HCO2)4H- Iso.2 *M*=1
E= -4038.835403
h -0.146391 0.000009 -1.018862
cu 1.377224 0.000002 -0.568729
o -3.755255 0.000012 -0.765321
cu -1.317923 -0.000003 0.045949
o 1.164745 1.972178 -0.572434
c 0.081739 2.506026 -0.224835
o -1.006495 1.976543 0.099475
o 1.164749 -1.972171 -0.572444
c 0.081752 -2.506024 -0.224828
o -1.006489 -1.976545 0.099467
c 3.622173 0.000001 0.918466
o 3.327080 0.000005 -0.327089
o 2.839524 -0.000006 1.865047
h 4.716688 0.000004 1.113345
c -3.853195 -0.000005 0.467077
o -2.879549 -0.000010 1.288236
h -4.859499 -0.000004 0.934282
h 0.094100 3.608662 -0.201437
h 0.094096 -3.608662 -0.201494

Cu2(HCO2)4H- Iso.2 *M*=3
E= -4038.821506
h -0.230730 0.000032 -1.261632
cu 1.280179 0.000001 -0.567989
o -3.822089 0.000209 -0.888591
cu -1.289779 -0.000038 0.015392
o 1.172147 1.984963 -0.326256
c 0.088229 2.520122 0.012372
o -1.009407 1.974386 0.279517
o 1.172198 -1.984943 -0.326283
c 0.088286 -2.520145 0.012293
o -1.009357 -1.974439 0.279471
c 3.748041 0.000054 0.587263
o 3.214823 0.000047 -0.581878
o 3.177297 0.000023 1.669151
h 4.858996 0.000101 0.546122
c -3.882481 -0.000059 0.340195
o -2.888515 -0.000106 1.145523
h -4.869429 0.000029 0.846904
h 0.105110 3.621901 0.088001
h 0.105212 -3.621919 0.087965

TS3
E= -4038.820754

H	-0.839467	0.075373	0.000000
Cu	0.373155	1.044866	0.000000
O	-2.956139	0.072216	0.000000
Cu	-0.564363	-1.515952	0.000000
O	0.318772	0.838045	1.916192
C	0.336853	-0.313495	2.435511
O	0.177320	-1.421322	1.890241
O	0.318772	0.838045	-1.916192
C	0.336853	-0.313495	-2.435511
O	0.177320	-1.421322	-1.890241
C	2.880710	2.182260	0.000000
O	1.656342	2.549713	0.000000
O	3.320660	1.034991	0.000000
H	3.594797	3.036801	0.000000
C	-3.272910	-1.126330	0.000000
O	-2.509266	-2.136250	0.000000
H	-4.358847	-1.367307	0.000000
H	0.519446	-0.314532	3.522700
H	0.519446	-0.314532	-3.522700

Cu2(HCO2)3(HCOOH) *M*=1
E= -4038.889527
H -2.839380 -0.002692 -0.427122
Cu 0.397061 -0.002242 -1.211916
O -3.738140 -0.004161 -0.955306
Cu 1.618196 0.002078 1.017536
O -1.449101 -0.001125 0.139781
C -1.309806 0.002473 1.402394
O -0.239955 0.003854 2.035828
O 0.885185 -1.906607 -1.360370
C 1.622244 -2.423205 -0.481822
O 2.113876 -1.891840 0.540703
C 1.620419 2.422131 -0.490338
O 0.884246 1.901597 -1.367339
O 2.111750 1.895067 0.534516
H 1.866457 3.488219 -0.651078
C -4.798916 -0.000641 -0.188186
O -4.839771 0.003439 1.022017
H -5.718493 -0.001904 -0.801699
H -2.248358 0.004546 1.984543
H 1.868952 -3.489744 -0.638533

Cartesian coordinates of optimized ions and molecules for Benchmarking (in Å, calculated at the noted level) along with electronic energies (in Hartree) including zero point energy

CO2 (M062x/6-31++g**)
E=-188.504123
O 0.000000 0.000000 1.162887
C 0.000000 0.000000 0.000000
O 0.000000 0.000000 -1.162887

CO2 (M062x/aug-cc-pVTZ)
E=-188.582240
O 0.000000 0.000000 1.155321
C 0.000000 0.000000 0.000000
O 0.000000 0.000000 -1.155321

CO2 (M062x/def2SVP)
E=-188.358325
O 0.000000 0.000000 1.156136
C 0.000000 0.000000 0.000000
O 0.000000 0.000000 -1.156136

CO2 (M062x/def2TZVP)
E=-188.584929
O 0.000000 0.000000 1.154702
C 0.000000 0.000000 0.000000
O 0.000000 0.000000 -1.154702

CO2 (B3LYP/6-31++g**)
E=-188.578828
O 0.000000 0.000000 1.169375
C 0.000000 0.000000 0.000000
O 0.000000 0.000000 -1.169375

CO2 (B3LYP/ aug-cc-pVTZ)
E=-188.651728
O 0.000000 0.000000 1.160476
C 0.000000 0.000000 0.000000
O 0.000000 0.000000 -1.160476

CO2 (B3LYP/ def2SVP)
E=-188.431261
O 0.000000 0.000000 1.163097
C 0.000000 0.000000 0.000000
O 0.000000 0.000000 -1.163097

CO2 (B3LYP/ def2TZVP)
E=-188.658232
O 0.000000 0.000000 1.159737
C 0.000000 0.000000 0.000000
O 0.000000 0.000000 -1.159737

CO2 (CCSD/6-31++g**)
E=-188.1198521
c 0.000000 0.000000 0.000000
o 0.000000 0.000000 1.171201
O 0.000000 0.000000 -1.171201

CO2 (CCSD/def2SVP)
E=- -187.9697375
c 0.000000 0.000000 0.000000
o 0.000000 0.000000 1.161601
o 0.000000 0.000000 -1.161601

CO2 (CCSD/def2TZVP)
E=-188.3219663
c 0.000000 0.000000 -0.000002
o 0.000000 0.000000 -1.158393
o 0.000000 0.000000 1.158395

CO2 (MP2/ 6-31++g**)
E=-188.106557
O 0.000000 0.000000 1.180711
C 0.000000 0.000000 0.000000
O 0.000000 0.000000 -1.180711

CO2 (MP2/ aug-cc-pVTZ)
E=-188.310147
O 0.000000 0.000000 1.170226
C 0.000000 0.000000 0.000000
O 0.000000 0.000000 -1.170226

CO2 (MP2/ def2SVP)
E=-187.955442
O 0.000000 0.000000 1.170396
C 0.000000 0.000000 0.000000
O 0.000000 0.000000 -1.170396

CO2 (MP2/ def2TZVP)
E=-188.303221
O 0.000000 0.000000 1.168597
C 0.000000 0.000000 0.000000
O 0.000000 0.000000 -1.168597

Cu(HCOO)H- (M062x/6-31++g**)
E=-1829.994146
Cu -1.088696 -0.074047 0.000000
O 2.012076 -0.892896 0.000000
C 1.750705 0.310838 0.000002
O 0.610705 0.885779 0.000000
H 2.603029 1.034324 -0.000005
H -2.517311 -0.695060 0.000000

Cu(HCOO)H- (M062x/ aug-cc-pVTZ)
E=-1830.340155
Cu -1.088708 -0.072964 0.000000
O 2.010089 -0.889463 0.000000
C 1.749976 0.308618 0.000001
O 0.614636 0.880742 0.000000
H 2.600249 1.028550 -0.000005
H -2.525361 -0.694520 0.000000

Cu(HCOO)H- (M062x/ def2SVP)
E=-1829.820662
Cu -1.067738 -0.071298 0.000001
O 1.951347 -0.904582 0.000000
C 1.724849 0.295588 -0.000005
O 0.610320 0.906390 -0.000004
H 2.606525 1.006423 0.000037
H -2.484569 -0.726757 0.000002

Cu(HCOO)H- (M062x/ def2TZVP)
E=-1830.294727
Cu -1.080682 -0.072140 0.000000
O 1.975490 -0.901988 0.000000
C 1.743417 0.300404 0.000001
O 0.622778 0.899811 0.000001
H 2.611424 1.002371 -0.000008
H -2.518303 -0.695334 -0.000001

Cu(HCOO)H- (B3LYP/6-31++g**)

E=-1830.190271
Cu -1.109214 -0.079057 0.000000
O 2.118834 -0.850803 -0.000001
C 1.760614 0.334027 0.000002
O 0.575197 0.829444 0.000000
H 2.552158 1.124500 -0.000005
H -2.500892 -0.665131 0.000001

Cu(HCOO)H- (B3LYP/ aug-cc-pVTZ)
E=-1830.527612
Cu -1.111138 -0.077905 0.000000
O 2.120084 -0.845630 0.000001
C 1.761391 0.330598 -0.000003
O 0.581409 0.822403 0.000000
H 2.549809 1.121233 0.000009
H -2.507094 -0.659767 0.000000

Cu(HCOO)H- (B3LYP/ def2SVP)
E=-1829.996365
Cu -1.096307 -0.077482 0.000000
O 2.077178 -0.859909 -0.000001
C 1.743688 0.319881 0.000003
O 0.579014 0.848039 0.000000
H 2.561289 1.110704 -0.000009
H -2.480062 -0.688058 0.000001

Cu(HCOO)H- (B3LYP/ def2TZVP)
E=-1830.483075
Cu -1.105124 -0.077812 0.000001
O 2.095151 -0.855049 -0.000004
C 1.755817 0.324703 0.000006
O 0.586345 0.838348 -0.000012
H 2.558775 1.105306 0.000049
H -2.497052 -0.663358 0.000015

Cu(HCOO)H- (CCSD/6-31++g**)
E=-1828.342356
c 1.742270 0.326005 0.000001
o 0.575049 0.868777 0.000000
cu -1.087396 -0.078985 0.000000
o 2.048208 -0.875423 0.000000
h 2.561071 1.075162 0.000000
h -2.466271 -0.687442 0.000000

Cu(HCOO)H- (CCSD/def2SVP)
E=-1828.138810
c 1.731722 0.307005 -0.000001
o 0.596421 0.887982 0.000001
cu -1.079356 -0.075068 0.000000
o 2.002542 -0.888360 0.000000
h 2.589048 1.045708 -0.000002
h -2.469757 -0.707733 -0.000001

Cu(HCOO)H- (CCSD/ def2TZVP)
E=-1828.828091
c 1.734173 0.316406 0.000000
o 2.023369 -0.875670 0.000000
o 0.586564 0.869624 0.000000
cu -1.082121 -0.076906 0.000000
h 2.567866 1.057314 0.000000
h -2.470860 -0.677099 0.000000

Cu(HCOO)H- (MP2/6-31++g**)/
E=-1828.392759
Cu -1.082661 -0.082155 0.000000
O 2.079359 -0.855675 -0.000003
C 1.728941 0.338708 0.000005
O 0.536846 0.846416 -0.000006
H 2.514703 1.121061 0.000019
H -2.420816 -0.696735 0.000009

Cu(HCOO)H- (MP2/ aug-cc-pVTZ)
E=-1829.073859
C 1.267468 -1.214186 0.000000
O 2.203293 -0.409213 0.000000
O 0.000000 -0.992916 0.000000
Cu -0.864578 0.652058 0.000000
H 1.501642 -2.298941 0.000000

Cu(HCOO)H- (MP2/ def2SVP)
E=-1828.165432
Cu -1.076882 -0.077770 0.000000
O 2.024566 -0.876029 0.000001
C 1.723555 0.315912 -0.000003
O 0.570901 0.875306 0.000001
H 2.558070 1.078826 0.000007
H -2.433569 -0.713164 -0.000002

Cu(HCOO)H- (MP2/ def2TZVP)
E=-1828.888426
Cu -1.077623 -0.080559 0.000000
O 2.058903 -0.853793 -0.000001
C 1.720733 0.330360 0.000002
O 0.544993 0.844968 -0.000004
H 2.517527 1.109249 0.000018
H -2.422005 -0.684604 0.000005

CuH2- (M062x/6-31++g**)
E=-1641.426591
Cu 0.000000 0.000000 -0.000004
H 0.000000 0.000000 -1.618004
H 0.000000 0.000000 1.618116

CuH2- (M062x/ aug-cc-pVTZ)
E=-1641.697806
Cu 0.000000 0.000000 -0.000004
H 0.000000 0.000000 -1.627330
H 0.000000 0.000000 1.627446

CuH2- (M062x/ def2SVP)
E=-1641.400241
Cu 0.000000 0.000000 -0.000003
H 0.000000 0.000000 -1.613530
H 0.000000 0.000000 1.613627

CuH2- (M062x/ def2TZVP)
E=-1641.650079
Cu 0.000000 0.000000 -0.000004
H 0.000000 0.000000 -1.626200
H 0.000000 0.000000 1.626308

CuH2- (B3LYP/6-31++g**)
E=-1641.556786
Cu 0.000000 0.000000 -0.000034
H 0.000000 0.000000 -1.571157
H 0.000000 0.000000 1.572152

CuH2- (B3LYP/ aug-cc-pVTZ)
E=-1641.826994
Cu 0.000000 0.000000 -0.000015
H 0.000000 0.000000 -1.572378
H 0.000000 0.000000 1.572808

CuH2- (B3LYP/ def2SVP)
E=-1641.509392

Cu 0.000000 0.000000 -0.000016
H 0.000000 0.000000 -1.572400
H 0.000000 0.000000 1.572869

CuH2- (B3LYP/ def2TZVP)
E=-1641.776027
Cu 0.000000 0.000000 -0.000034
H 0.000000 0.000000 -1.571157
H 0.000000 0.000000 1.572152

CuH2- (CCSD/6-31++g**)
E=-1640.182432
cu 0.000000 0.000000 -0.000002
h 0.000000 0.000000 -1.564236
h 0.000000 0.000000 1.564303

CuH2- (CCSD/def2SVP)
E=-1640.132654
cu 0.000000 0.000920 0.000000
h 1.580048 -0.008835 0.000000
h -1.580048 -0.017832 0.000000

CuH2- (CCSD/ def2TZVP)
E=-1640.477483
cu 0.000000 0.000000 -0.000002
h 0.000000 0.000000 -1.565791
h 0.000000 0.000000 1.565852

CuH2- (MP2/6-31++g**)
E=-1640.232483
Cu 0.000000 0.000000 -0.000002
H 0.000000 0.000000 -1.526447
H 0.000000 0.000000 1.526492

CuH2- (MP2/ aug-cc-pVTZ)
E=-1640.711731
Cu 0.000000 0.000000 -0.000002
H 0.000000 0.000000 -1.525367
H 0.000000 0.000000 1.525415

CuH2- (MP2/ def2SVP)
E=-1640.159427
Cu 0.000000 0.000000 -0.000002
H 0.000000 0.000000 -1.551204
H 0.000000 0.000000 1.551270

CuH2- (MP2/ def2TZVP)
E=-1640.533030
Cu 0.000000 0.000000 -0.000002
H 0.000000 0.000000 -1.523571
H 0.000000 0.000000 1.523619

Cu(HCOO)2- (M062x/6-31++g**)
E=-2018.552099
C -2.756153 -0.308595 -0.000004
O -2.928232 0.908978 0.000020
O -1.650673 -0.956389 -0.000027
Cu -0.000001 0.000010 -0.000006
O 1.650684 0.956386 0.000004
C 2.756156 0.308579 0.000013
O 2.928221 -0.908995 0.000022
H -3.648229 -0.976147 -0.000032
H 3.648239 0.976122 0.000012

Cu(HCOO)2- (M062x/ aug-cc-pVTZ)
E=-2018.973106
C -2.752731 -0.302983 -0.000009
O -2.912366 0.910604 -0.000289
O -1.658081 -0.957786 0.000130
Cu 0.000011 -0.000363 0.000104
O 1.657651 0.957848 0.000139
C 2.752625 0.303577 -0.000067
O 2.912889 -0.909926 -0.000275
H -3.649640 -0.957890 -0.000148
H 3.649202 0.958934 -0.000047

Cu(HCOO)2- (M062x/ def2SVP)
E=-2018.230429
C -2.718391 -0.290096 0.000277
O -2.843262 0.924090 0.001130
O -1.646827 -0.979326 -0.000486
Cu -0.000012 0.000372 -0.000526
O 1.647281 0.979252 -0.000435
C 2.718495 0.289484 0.000365
O 2.842720 -0.924769 0.001122
H -3.646318 -0.931558 0.000387
H 3.646757 0.930464 0.000376

Cu(HCOO)2- (M062x/ def2TZVP)
E=-2018.928610
C -2.743070 -0.296658 -0.000003
O -2.885150 0.917800 -0.000241
O -1.659567 -0.968567 0.000102
Cu 0.000012 -0.000366 0.000091
O 1.659124 0.968633 0.000112
C 2.742962 0.297257 -0.000062
O 2.885687 -0.917122 -0.000227
H -3.650946 -0.939770 -0.000159
H 3.650504 0.940835 -0.000058

Cu(HCOO)2- (B3LYP/6-31++g**)
E=-2018.811216
C -2.780343 -0.332312 -0.000025
O -3.044083 0.873691 -0.000002
O -1.626900 -0.911666 -0.000014
Cu 0.000003 -0.000067 0.000002
O 1.626827 0.911673 0.000021
C 2.780323 0.332419 0.000012
O 3.044171 -0.873558 -0.000004
H -3.616998 -1.068645 -0.000011
H 3.616909 1.068829 0.000020

Cu(HCOO)2- (B3LYP/ aug-cc-pVTZ)
E=-2019.215178
C -2.783419 -0.328481 0.000000
O -3.046801 0.869301 0.000061
O -1.636257 -0.906061 -0.000044
Cu -0.000009 0.000162 -0.000021
O 1.636426 0.906039 -0.000015
C 2.783472 0.328220 0.000021
O 3.046599 -0.869616 0.000062
H -3.618562 -1.063396 -0.000033
H 3.618765 1.062964 0.000011

Cu(HCOO)2- (B3LYP/ def2SVP)
E=-2018.468606
C -2.760824 -0.317562 -0.000042
O -2.996984 0.883196 -0.000108
O -1.631056 -0.928268 0.000037
Cu 0.000003 -0.000074 0.000042
O 1.630972 0.928275 0.000070
C 2.760802 0.317682 -0.000014
O 2.997086 -0.883051 -0.000108
H -3.625331 -1.048213 -0.000027
H 3.625233 1.048423 0.000009

Cu(HCOO)2- (B3LYP/ def2TZVP)
E=-2019.175455
C -2.774974 -0.323672 0.000161
O -3.026758 0.874543 0.000263
O -1.635646 -0.914947 -0.000086
Cu -0.000014 -0.000151 -0.000173
O 1.635446 0.914870 -0.000094
C 2.774920 0.323973 0.000146
O 3.027078 -0.874205 0.000264
H -3.618739 -1.052843 0.000155
H 3.618513 1.053326 0.000242

Cu(HCOO)2- (CCSD/6-31++g**)
E=-2016.490964
-2.748127 -0.322779 0.000234
-1.616205 -0.945808 0.000046
0.000016 -0.000292 0.000129
1.615870 0.945848 0.000235
2.748033 0.323254 -0.000050
2.960048 -0.895956 -0.000402
-2.959655 0.896517 -0.000511
-3.611436 -1.015258 0.000150
3.611067 1.016074 0.000051

Cu(HCOO)2- (MP2/6-31++g**)
E=-2016.541311
C -2.738337 -0.337932 0.000001
O -3.001132 0.875604 0.000062
O -1.576170 -0.923223 -0.000044
Cu -0.000012 -0.000252 -0.000024
O 1.575884 0.923210 -0.000015
C 2.738268 0.338348 0.000027
O 3.001558 -0.875081 0.000061
H -3.565985 -1.071464 -0.000010
H 3.565627 1.072203 0.000030

Cu(HCOO)2- (MP2/ aug-cc-pVTZ)
E=-2017.424446
C -2.731113 -0.335232 -0.000038
O -2.997130 0.867345 0.000019
O -1.575345 -0.912009 -0.000029
Cu -0.000006 0.000081 -0.000002
O 1.575433 0.911999 0.000014
C 2.731140 0.335102 0.000020
O 2.997031 -0.867504 0.000012
H -3.557154 -1.071605 0.000023
H 3.557259 1.071388 0.000038

Cu(HCOO)2- (MP2/ def2SVP)
E=-2016.158659
C -2.730984 -0.313124 -0.000052
O -2.935487 0.897995 -0.000094
O -1.615938 -0.952024 0.000029
Cu -0.000024 0.000436 0.000040
O 1.616441 0.951958 0.000067
C 2.731120 0.312416 -0.000011
O 2.934900 -0.898826 -0.000096
H -3.612877 -1.014172 -0.000022
H 3.613426 1.012946 0.000004

Cu(HCOO)2- (MP2/ def2TZVP)
E=-2017.230288
C -2.730280 -0.330968 -0.000036
O -2.988736 0.870418 -0.000007
O -1.581956 -0.916451 -0.000016
Cu -0.000002 0.000022 0.000005
O 1.581979 0.916447 0.000025
C 2.730287 0.330933 0.000016
O 2.988711 -0.870460 -0.000013
H -3.565215 -1.063675 0.000022
H 3.565242 1.063618 0.000041

CO2 (BMK/6-31++g**)
E=-188.497946
O 0.000000 0.000000 1.162068
C 0.000000 0.000000 0.000000
O 0.000000 0.000000 -1.162068

CO2 (BMK/AUG-cc-pVTZ)
E=-188.561725
O 0.000000 0.000000 1.153643
C 0.000000 0.000000 0.000000
O 0.000000 0.000000 -1.153643

CO2 (BMK/ def2SVP)
E=-188.346540
O 0.000000 0.000000 1.155907
C 0.000000 0.000000 0.000000
O 0.000000 0.000000 -1.155907

CO2 (BMK/ def2TZVP)
E=-188.569887
O 0.000000 0.000000 1.152831
C 0.000000 0.000000 0.000000
O 0.000000 0.000000 -1.152831

CO2 (M06/6-31++g**)
E=-188.497672
O 0.000000 0.000000 1.164154
C 0.000000 0.000000 0.000000
O 0.000000 0.000000 -1.164154

CO2 (M06/ AUG-cc-pVTZ)
E=-188.560537
O 0.000000 0.000000 1.154646
C 0.000000 0.000000 0.000000
O 0.000000 0.000000 -1.154646

CO2 (M06L/6-31++g**)
E=-188.569769
O 0.000000 0.000000 1.168426
C 0.000000 0.000000 0.000000
O 0.000000 0.000000 -1.168426

CO2 (M06L/ AUG-cc-pVTZ)
E=-188.630233
O 0.000000 0.000000 1.160201
C 0.000000 0.000000 0.000000
O 0.000000 0.000000 -1.160201

CO2 (M06L/ def2SVP)
E=-188.422166
O 0.000000 0.000000 1.162160
C 0.000000 0.000000 0.000000
O 0.000000 0.000000 -1.162160

CO2 (M06L/ def2TZVP)
E=-188.641719
O 0.000000 0.000000 1.159224
C 0.000000 0.000000 0.000000
O 0.000000 0.000000 -1.159224

CO2 (M06/ def2SVP)
E=-188.348928
O 0.000000 0.000000 1.158638

C 0.000000 0.000000 0.000000
O 0.000000 0.000000 -1.158638

CO2 (M06/ def2TZVP)
E=-188.572561
O 0.000000 0.000000 1.154117
C 0.000000 0.000000 0.000000
O 0.000000 0.000000 -1.154117

Cu(HCOO)H- (BMK/6-31++g**)
E=-1829.270504
Cu -1.095393 -0.077212 0.000000
O 2.087171 -0.851043 0.000003
C 1.743310 0.329997 -0.000010
O 0.570022 0.829321 0.000002
H 2.544491 1.111960 0.000023
H -2.495484 -0.679012 -0.000004

Cu(HCOO)H- (BMK/ AUG-cc-pVTZ)
E=-1829.597266
Cu -1.098787 -0.076062 0.000000
O 2.096324 -0.842226 0.000001
C 1.745651 0.329009 -0.000002
O 0.573870 0.815738 0.000001
H 2.537432 1.114377 0.000002
H -2.508072 -0.670728 -0.000001

Cu(HCOO)H- (BMK/ def2SVP)
E=-1829.078895
Cu -1.081358 -0.075548 0.000000
O 2.044500 -0.860316 -0.000001
C 1.725921 0.317526 0.000002
O 0.571801 0.847015 -0.000003
H 2.551574 1.095736 0.000014
H -2.478133 -0.703584 0.000004

Cu(HCOO)H- (BMK/ def2TZVP)
E=-1829.552966
Cu -1.092331 -0.075563 0.000000
O 2.063269 -0.857024 -0.000001
C 1.741129 0.321020 0.000002
O 0.583745 0.839209 -0.000001
H 2.554000 1.088171 0.000000
H -2.499275 -0.680439 0.000002

Cu(HCOO)H- (M06/6-31++g**)
E=-1830.013310
Cu -1.073101 -0.076295 0.000000
O 2.004958 -0.883061 0.000000
C 1.723429 0.315013 -0.000001
O 0.577712 0.880299 -0.000001
H 2.564151 1.059155 0.000007
H -2.446152 -0.714587 0.000001

Cu(HCOO)H- (M06/ AUG-cc-pVTZ)
E=-1830.323267
Cu -1.073726 -0.075414 0.000000
O 2.008740 -0.874777 0.000000
C 1.721148 0.312336 -0.000001
O 0.579283 0.870000 -0.000001
H 2.555516 1.061545 0.000008
H -2.448542 -0.710335 0.000001

Cu(HCOO)H- (M06L/6-31++g**)
E=-1830.059530
Cu -1.078504 -0.077559 0.000000
O 2.027890 -0.878059 0.000000
C 1.727620 0.318672 0.000002
O 0.572226 0.875189 0.000001
H 2.556865 1.077301 -0.000009
H -2.446894 -0.717172 0.000000

Cu(HCOO)H- (M06L/ AUG-cc-pVTZ)
E=-1830.380167
Cu -1.079041 -0.076326 0.000000
O 2.026187 -0.873275 0.000000
C 1.728728 0.317000 0.000001
O 0.576788 0.866477 0.000001
H 2.554179 1.073349 -0.000011
H -2.458159 -0.707521 -0.000001

Cu(HCOO)H- (M06L/ def2SVP)
E=-1829.862576
Cu -1.057911 -0.074868 0.000000
O 1.959000 -0.896507 -0.000003
C 1.705840 0.303115 0.000009
O 0.579390 0.902532 -0.000004
H 2.571875 1.040295 -0.000004
H -2.434626 -0.736017 0.000009

Cu(HCOO)H- (M06L/ def2TZVP)
E=-1830.343761
Cu -1.070535 -0.075625 0.000000
O 1.992367 -0.885906 0.000002
C 1.720523 0.309269 -0.000008
O 0.583008 0.886503 0.000002
H 2.565011 1.049564 0.000011
H -2.445651 -0.716824 -0.000006

Cu(HCOO)H- (M06/ def2SVP)
E=-1829.805828
Cu -1.061590 -0.074467 0.000000
O 1.965922 -0.891791 -0.000001
C 1.708208 0.302498 -0.000001
O 0.583285 0.895595 -0.000003
H 2.574167 1.042359 0.000018
H -2.430955 -0.728234 0.000003

Cu(HCOO)H- (M06/ def2TZVP)
E=-1830.289739
Cu -1.070945 -0.075394 0.000000
O 1.993883 -0.880957 -0.000001
C 1.718169 0.307456 0.000002
O 0.585233 0.880507 -0.000001
H 2.563763 1.050274 -0.000001
H -2.448310 -0.704985 0.000002

CuH2- (BMK/6-31++g**)
E=-1640.714205
Cu 0.000000 0.000000 -0.000003
H 0.000000 0.000000 -1.583913
H 0.000000 0.000000 1.583998

CuH2- (BMK/ AUG-cc-pVTZ)
E=-1640.981786
Cu 0.000000 0.000000 -0.000003
H 0.000000 0.000000 -1.589025
H 0.000000 0.000000 1.589102

CuH2- (BMK/ def2SVP)
E=-1640.673131
Cu 0.000000 0.000000 -0.000002
H 0.000000 0.000000 -1.584617
H 0.000000 0.000000 1.584672

CuH2- (BMK/ def2TZVP)

E=-1640.929992
Cu 0.000000 0.000000 -0.000003
H 0.000000 0.000000 -1.590645
H 0.000000 0.000000 1.590724

CuH2- (M06/6-31++g**)
E=-1641.465630
Cu 0.000000 0.000000 -0.000014
H 0.000000 0.000000 -1.572847
H 0.000000 0.000000 1.573264

CuH2- (M06/ AUG-cc-pVTZ)
E=-1641.720493
Cu 0.000000 0.000000 -0.000014
H 0.000000 0.000000 -1.572677
H 0.000000 0.000000 1.573086

CuH2- (M06L/6-31++g**)
E=-1641.444056
Cu 0.000000 0.000000 -0.000034
H 0.000000 0.000000 -1.571157
H 0.000000 0.000000 1.572152

CuH2- (M06L/ AUG-cc-pVTZ)
E=-1641.708772
Cu 0.000000 0.000000 -0.000003
H 0.000000 0.000000 -1.575670
H 0.000000 0.000000 1.575751

CuH2- (M06L/ def2SVP)
E=-1641.388719
Cu 0.000000 0.000000 -0.000003
H 0.000000 0.000000 -1.578819
H 0.000000 0.000000 1.578905

CuH2- (M06L/ def2TZVP)
E=-1641.662375
Cu 0.000000 0.000000 -0.000003
H 0.000000 0.000000 -1.577047
H 0.000000 0.000000 1.577132

CuH2- (M06/ def2SVP)
E=-1641.401299
Cu 0.000000 0.000000 -0.000015
H 0.000000 0.000000 -1.572467
H 0.000000 0.000000 1.572896

CuH2- (M06/ def2TZVP)
E=-1641.675120
Cu 0.000000 0.000000 -0.000014
H 0.000000 0.000000 -1.573397
H 0.000000 0.000000 1.573809

Cu(HCOO)2- (BMK/6-31++g**)
E=-2017.815007
C -2.756816 -0.328541 0.000132
O -3.006172 0.873302 0.000846
O -1.616236 -0.910150 -0.000409
Cu 0.000001 -0.000034 -0.000313
O 1.616198 0.910152 -0.000373
C 2.756805 0.328597 0.000178
O 3.006219 -0.873233 0.000841
H -3.604487 -1.053890 -0.000021
H 3.604441 1.053988 0.000003

Cu(HCOO)2- (BMK/ AUG-cc-pVTZ)
E=-2018.200695
C -2.762333 -0.325765 0.000031
O -3.012343 0.868350 0.000264
O -1.626300 -0.902110 -0.000137
Cu 0.000012 -0.000187 -0.000095
O 1.626098 0.902135 -0.000107
C 2.762272 0.326067 0.000059
O 3.012578 -0.867986 0.000262
H -3.606256 -1.049252 -0.000035
H 3.606016 1.049761 0.000003

Cu(HCOO)2- (BMK/ def2SVP)
E=-2017.471891
C 2.742287 -0.313099 0.015678
O 2.957180 0.884532 0.082324
O 1.627061 -0.926870 -0.039568
Cu 0.000000 0.000001 -0.029843
O -1.627062 0.926871 -0.039577
C -2.742287 0.313098 0.015647
O -2.957178 -0.884534 0.082275
H 3.619387 -1.024910 -0.003052
H -3.619388 1.024908 -0.003085

Cu(HCOO)2- (BMK/ def2TZVP)
E=-2018.162442
C -2.755786 -0.321514 0.000011
O -2.995124 0.873194 0.000054
O -1.627017 -0.911149 -0.000038
Cu -0.000027 0.000050 -0.000029
O 1.627051 0.911085 -0.000017
C 2.755817 0.321453 0.000041
O 2.995160 -0.873257 0.000050
H -3.607634 -1.039061 0.000044
H 3.607666 1.038998 0.000087

Cu(HCOO)2- (M06/6-31++g**)
E=-2018.548133
Cu 0.000000 0.000000 -0.067161
O 1.859142 0.004295 -0.067161
O -1.859142 -0.004295 -0.067161
C 2.495676 -1.110191 -0.067162
O 2.048805 -2.253972 -0.067163
H 3.603595 -0.959222 -0.067161
C -2.495676 1.110191 -0.067162
H -3.603595 0.959222 -0.067161
O -2.048805 2.253972 -0.067163

Cu(HCOO)2- (M06/ AUG-cc-pVTZ)
E=-2018.913659
C -2.716890 -0.313152 0.000078
O -2.926457 0.887153 0.000499
O -1.603328 -0.936956 -0.000259
Cu 0.000006 -0.000123 -0.000181
O 1.603186 0.936973 -0.000226
C 2.716853 0.313351 0.000111
O 2.926626 -0.886916 0.000498
H -3.584782 -1.016255 -0.000011
H 3.584624 1.016603 0.000022

Cu(HCOO)2- (M06L/6-31++g**)
E=-2018.662137
C -2.721054 -0.316340 0.020493
O -2.929387 0.894609 0.096590
O -1.599889 -0.948028 -0.046143
Cu -0.000001 -0.000036 -0.036516
O 1.599840 0.948031 -0.046154
C 2.721036 0.316396 0.020487
O 2.929461 -0.894536 0.096594
H -3.589632 -1.021169 0.002980
H 3.589567 1.021284 0.003001

Cu(HCOO)2- (M06L/ AUG-cc-pVTZ)
E=-2019.039642
C -2.725044 -0.314424 0.000182
O -2.930860 0.892434 0.001073
O -1.608071 -0.943289 -0.000536
Cu 0.000003 -0.000001 -0.000388
O 1.608071 0.943296 -0.000501
C 2.725041 0.314423 0.000221
O 2.930850 -0.892435 0.001071
H -3.592277 -1.015739 -0.000016
H 3.592277 1.015735 0.000011

Cu(HCOO)2- (M06L/ def2SVP)
E=-2018.325079
C -2.698154 -0.299299 -0.000005
O -2.854226 0.915476 -0.000129
O -1.609963 -0.974069 0.000057
Cu 0.000000 -0.000006 0.000035
O 1.609956 0.974068 0.000078
C 2.698154 0.299308 -0.000006
O 2.854236 -0.915465 -0.000127
H -3.607539 -0.973680 -0.000023
H 3.607532 0.973699 0.000039

Cu(HCOO)2- (M06L/ def2TZVP)
E=-2019.013024
C -2.717145 -0.309647 0.000118
O -2.911277 0.897880 0.000857
O -1.608693 -0.952274 -0.000450
Cu 0.000004 -0.000011 -0.000292
O 1.608683 0.952284 -0.000411
C 2.717140 0.309661 0.000165
O 2.911280 -0.897864 0.000855
H -3.593997 -1.003898 -0.000032
H 3.593985 1.003919 -0.000012

Cu(HCOO)2- (M06/ def2SVP)
E=-2018.196687
Cu 0.000000 0.000000 -0.032901
O 1.869434 0.017784 -0.025418
O -1.869434 -0.017784 -0.025418
C 2.481080 -1.102151 -0.072935
O 2.022550 -2.231269 -0.125257
H 3.606073 -0.964446 -0.062168
C -2.481080 1.102151 -0.072935
H -3.606073 0.964446 -0.062168
O -2.022550 2.231269 -0.125257

Cu(HCOO)2- (M06/ def2TZVP)
E=-2018.890366
C -2.711962 -0.309431 0.000089
O -2.915840 0.890272 0.000601
O -1.604516 -0.942850 -0.000319
Cu 0.000010 -0.000209 -0.000206
O 1.604275 0.942880 -0.000286
C 2.711899 0.309770 0.000119
O 2.916127 -0.889874 0.000600
H -3.586369 -1.010232 -0.000042
H 3.586105 1.010821 -0.000007

Cartesian coordinates of optimized $Cu^{+}(H_2O)_n$ and $CuOH^{+}(H_2O)_n$ (in Å) along with electronic energies (in Hartree) including zero point energy

Calculated at the BMK/def2TZVP level

Cu+
E=-1639.422366
Cu 0.000000 0.000000 0.000000

Cu+.H2O
E=-1715.889165
Cu 0.000010 0.527156 0.000000
O 0.000010 -1.416431 0.000000
H 0.782795 -1.978313 0.000000
H -0.783174 -1.977758 0.000000

Cu+.(H2O)2
E=-1792.356370
Cu 0.000000 0.000023 0.000007
O -1.911371 0.000027 0.000069
H -2.469641 -0.554513 0.553769
H -2.469687 0.553926 -0.554227
O 1.911371 0.000075 -0.000119
H 2.469744 0.553813 0.554280
H 2.469582 -0.554722 -0.553621

Cu+.(H2O)3
E=-1868.777003
Cu 0.150178 -0.172446 -0.000676
O -0.986398 1.701188 0.003507
H -1.059649 2.287123 0.762882
H -1.133222 2.241380 -0.778806
O -1.563652 -1.203061 -0.010064
H -1.718268 -2.150528 0.020652
H -2.408360 -0.748710 0.054263
O 2.124442 -0.066493 0.009222
H 2.723288 -0.818073 0.008047
H 2.645928 0.736675 -0.068756

Cu+.(H2O)2
E=-1945.192217
O 1.015924 -1.020644 -1.359275
Cu -0.336488 0.036805 0.030572
O -2.347677 -0.022122 -0.002780
O 1.153101 -0.692622 1.462099
O 1.142596 1.620855 -0.204805
H 1.578521 -0.366408 -1.787277
H 1.733493 1.570149 0.554479
H 1.023230 2.551779 -0.412549
H -2.884861 -0.574134 0.571251
H -2.907898 0.680542 -0.342192
H 1.062062 -0.958209 2.381475
H 1.656095 -1.380225 1.011932
H 0.785949 -1.674574 -2.025627

CuOH+
E=-1715.197838
O 0.020649 1.381012 0.000000
Cu 0.020649 -0.448648 0.000000
H -0.763998 1.962693 0.000000

CuOH+H2O
E=-1791.678402
O -1.806096 -0.005124 0.000198
Cu 0.081796 0.012908 -0.000180
O 1.798346 -0.115955 0.000300
H -2.379686 0.771769 0.000496
H -2.351507 -0.802459 0.000344
H 2.421106 0.624982 0.000395

CuOH+(H2O)2
E=-1868.127122
O 1.351106 1.270822 0.016420
Cu -0.211152 0.061582 0.020531
O 0.974893 -1.539350 -0.031478
O -1.936019 0.127611 -0.121423
H 1.255407 2.216776 0.178824
H 2.102836 1.139202 -0.572790
H -2.570802 0.109249 0.600183
H 1.593600 -1.758287 0.674906
H 0.622535 -2.365495 -0.384671

CuOH+(H2O)3
E=-1944.561772
O 0.004483 -1.828888 -0.129096
Cu 0.000104 -0.008582 -0.044288
O -2.000127 -0.132332 0.178558
O 2.000784 -0.123395 0.178482
O -0.005271 1.989259 -0.115663
H 0.004669 -2.236812 -0.998794
H 2.565007 0.240359 0.869116
H 2.061806 -1.093834 0.202501
H 0.774076 2.491237 -0.378365
H -0.786907 2.485982 -0.381579
H -2.056480 -1.103088 0.201975
H -2.564140 0.227870 0.871254

CuOH+(H2O)4
E=-2020.987714
O -1.875394 -1.257685 0.193421
Cu -0.525855 -0.068052 -0.044347
O 0.972061 -1.396327 -0.152211
O 0.827317 1.397193 -0.067744
O -2.043134 1.273676 0.088348
H -1.934214 -2.057753 -0.329044
H 1.776114 1.193447 0.026480
H 0.635098 2.262463 0.305034
H -2.333651 1.869237 -0.609642
H -2.717115 0.574614 0.192984
H 1.901400 -1.159555 0.004771
H 0.775882 -2.264067 0.215209
O 3.294226 0.136071 0.096621
H 3.859649 0.164194 0.876500
H 3.886009 0.167496 -0.663710

Calculated at the B3LYP/def2TZVP level

Cu+
E=-1640.246610
Cu 0.000000 0.000000 0.000000

Cu+H2O
E=-1716.753476
Cu 0.000010 0.527649 0.000000
O 0.000010 -1.417359 0.000000
H 0.785048 -1.981736 0.000000
H -0.785401 -1.981219 0.000000

Cu+(H2O)2
E=-1793.259876
Cu 0.000000 0.000000 0.002318

O 0.000000 1.912784 -0.015591
O 0.000000 -1.912784 -0.015591
H -0.620746 2.460661 -0.512589
H 0.483796 2.473862 0.603710
H 0.620746 -2.460661 -0.512589
H -0.483796 -2.473862 0.603710

Cu+(H2O)3
E=-1869.722636
Cu -0.149418 -0.247507 0.005669
O 0.888596 1.770872 -0.014701
H 0.833531 2.403432 -0.742098
H 1.106084 2.282824 0.774632
O 1.638442 -1.084952 0.040479
H 1.854765 -2.011485 -0.116679
H 2.401838 -0.545654 -0.200725
O -2.100448 -0.046138 -0.045050
H -2.713144 -0.791588 -0.037270
H -2.562667 0.721925 0.311920

Cu+(H2O)4
E=-1946.181101
Cu -0.282706 0.141737 0.011675
O 0.721099 -1.658878 -0.910295
H 0.252000 -2.464522 -1.159957
H 1.310284 -1.449255 -1.646023
O 1.205383 1.400842 -0.846566
H 1.062534 2.198794 -1.369231
H 1.902541 1.600356 -0.208340
O -2.305097 0.034180 0.094979
H -2.782644 -0.080560 0.925181
H -2.871513 0.552873 -0.488626
O 1.203721 -0.195359 1.606918
H 1.122719 -0.046240 2.556602
H 1.601718 -1.068109 1.491543

CuOH+
E=-1716.066690
O 0.021346 1.367922 0.000000
Cu 0.021346 -0.443473 0.000000
H -0.789819 1.917339 0.000000

CuOH+H2O
E=-1792.585160
O -1.824119 -0.003018 0.000147
Cu 0.078423 0.010967 -0.000128
O 1.833262 -0.115999 0.000210
H -2.398479 0.776345 0.000318
H -2.374565 -0.799836 0.000250
H 2.425648 0.657578 0.000294

CuOH+(H2O)2
E=-1869.068163
O 1.148328 -1.460199 0.030594
Cu -0.220506 0.024071 -0.016939
O 1.240845 1.390209 -0.025005
O -1.978955 0.020370 0.120218
H 1.719076 -1.644805 -0.728521
H 0.909463 -2.310444 0.426558
H -2.580035 0.019958 -0.639471
H 1.050300 2.333382 -0.122676
H 2.014132 1.300809 0.548888

CuOH+(H2O)3
E=-1945.539158
O 0.622587 -1.759601 0.054602
Cu 0.006267 -0.037430 -0.029845
O -1.951491 -0.582900 0.116898
O -0.577001 1.904156 -0.044095
O 1.985052 0.445972 -0.012409
H 0.400465 -2.330209 -0.691881
H -1.321321 2.257697 0.459394
H -0.037771 2.640065 -0.360303
H 2.423976 0.926105 0.703242
H 2.300426 -0.478801 0.005888
H -1.985605 -1.500596 0.428334
H -2.595098 -0.489796 -0.599120

CuOH+(H2O)4
E=-2022.007413
O -1.827469 -1.302518 0.261957
Cu -0.518271 -0.055830 -0.052732
O 0.958613 -1.420006 -0.215947
O 0.837572 1.430466 -0.093800
O -2.074838 1.266532 0.095873
H -1.989711 -1.946875 -0.437342
H 1.783505 1.209903 0.031885
H 0.646012 2.282137 0.316907
H -2.392496 1.805554 -0.641056
H -2.726051 0.551022 0.247901
H 1.879776 -1.177060 -0.003987
H 0.726010 -2.252150 0.217362
O 3.278817 0.130000 0.137051
H 3.832395 0.151377 0.929707
H 3.888871 0.159362 -0.613230
